# Scaling Cultured Meat: Challenges and Solutions for Affordable Mass Production

**DOI:** 10.1111/1541-4337.70221

**Published:** 2025-07-09

**Authors:** Huiwen Gu, Yan Kong, Dejian Huang, Youfa Wang, Vijaya Raghavan, Jin Wang

**Affiliations:** ^1^ Key Laboratory of Environmental Medicine and Engineering, and Department of Nutrition and Food Hygiene, Ministry of Education, School of Public Health Southeast University Nanjing China; ^2^ National University of Singapore (Suzhou) Research Institute Suzhou Jiangsu China; ^3^ Department of Food Science and Technology National University of Singapore Singapore Singapore; ^4^ Department of Bioresource Engineering, Faculty of Agricultural and Environmental Sciences McGill University Quebec Canada

**Keywords:** bioengineering, cell sourcing, cell medium, cultured meat, future food, new protein source, scaffold

## Abstract

As the global population grows and meat consumption increases, the demand for sustainable and efficient food systems becomes urgent. Cultured meat (CM) has emerged as a promising alternative to conventional meat, offering potential benefits in environmental conservation, resource efficiency, and animal welfare. Although the cost of CM has dropped dramatically—from $2.3 million/kg for the first cultured beef burger to $63/kg—it remains prohibitively expensive and confined to small‐scale production. Recent advancements in areas, such as cell density, cell doubling times, and bioreactor efficiency, have shown promise in further reducing costs. Thus, transformative innovations in all aspects of CM production will contribute to achieving price parity with conventional meat. This review explores the four core technologies underpinning CM production: cell line development, serum‐free media, scaffold fabrication, and bioreactor design, with a focus on achieving economical, large‐scale production through their interdependence and integration. These technologies converge around three key breakthroughs: engineering genetically stable, highly expandable, and functionalized cell lines to minimize reliance on tissue sampling and expensive growth factors; utilizing plant‐based substitutes and recombinant protein alternatives to reduce the costs of media and scaffolds while enhancing biocompatibility; and optimizing bioreactors to provide dynamic environmental control, enabling high‐density cell cultures at scale. By synthesizing recent advancements and addressing critical challenges, this review outlines a roadmap for cost‐effective, industrial‐scale CM production. It provides strategies to reduce costs, improve scalability, and contribute to global food security, ultimately establishing CM as a viable and sustainable alternative to conventional meat production.

## 1 Introduction

By 2050, the global population is projected to increase by 38.6%, driving a 33.3% rise in global meat consumption (Gu et al. 2023). Although existing agricultural models could theoretically meet this demand, inefficiencies in production, resource losses, and unequal access to food systems severely undermine their effectiveness (Berners‐Lee et al. 2018). Transitioning to more sustainable practices, such as agroecological models and sustainable intensification, could enhance productivity on current farmland and mitigate environmental degradation. However, these approaches face significant socio‐economic and policy barriers that limit their scalability (Sijpestijn et al. 2022). In this context, cultured meat (CM)—a technology that produces meat by growing animal cells in vitro—has emerged as a potential solution, promising to reduce environmental impact, conserve resources, and improve animal welfare (Post et al. 2020). Nevertheless, the feasibility of CM production at scale remains uncertain, as current lifecycle assessments and economic projections rely heavily on unverified assumptions (Lynch and Pierrehumbert 2019; Risner et al. 2020). Concerns persist about the scalability, affordability, and energy demands of CM production, as industrial systems may require higher energy inputs than biological processes (Escobar 2021). The consequent high production costs are also a factor in the acceptance of CM by consumers. Surveys have indicated that CM acceptance could increase by 27% and 55% if prices matched or fell below those of conventional meat (Chriki et al. 2024). This highlights the need for empirical validation and technical breakthroughs to fully realize CM's economic potential. Although factors like labor and capital costs also impact CM pricing (Garrison et al. 2022), this review focuses on advancements in manufacturing technologies to lower costs and enhance scalability.

The financial expense of CM has decreased considerably since the pioneering advent of the cultured beef burger in 2013, which was priced at $2.3 million/kg (Post 2014). Despite such advances, the scalability and affordability of CM remain inadequate for effective competition with conventional meat products (Park et al. 2024; Lee et al. 2025). For instance, the production of 42 sheets of CM, each measuring 2 cm^2^, incurred a cost of $301.15 (Park et al. 2021), whereas estimates for large‐scale CM production suggest a cost of approximately $63/kg (Garrison et al. 2022). Achieving price parity with conventional meat would require transformative innovations across all aspects of CM production. Breakthroughs in cell densities, doubling times, and bioreactor efficiency have demonstrated the potential for cost reductions, with one optimized system lowering costs from $437,000 to $1.95/kg (Risner et al. 2020).

**FIGURE 1:**
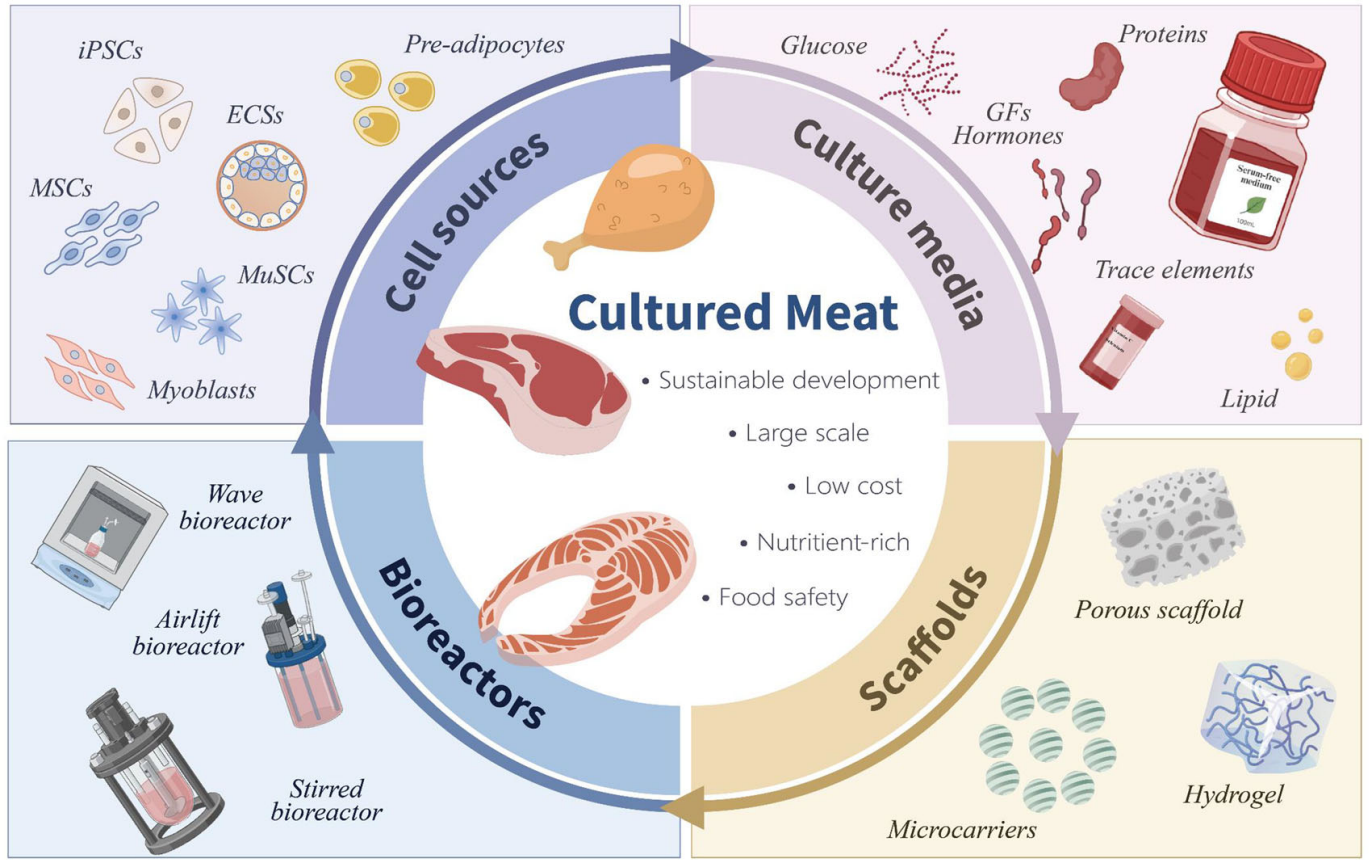
Four key synergistic technologies to achieve sustainable, large‐scale, low‐cost, nutritious, and safe cultured meat. Cell sources, including induced pluripotent stem cells (iPSCs), embryonic stem cells (ESCs), mesenchymal stem cells (MSCs), muscle satellite cells (MuSCs), myoblasts, and pre‐adipocytes. Cultured media blending glucose, growth factors (GFs), hormones, proteins, trace elements, lipid, and so on. Three types of scaffolds involve porous scaffold, microcarriers, and hydrogel. Scalable bioreactor systems encompassing wave bioreactors, airlift bioreactor, stirred bioreactor, and so on.

CM production is contingent on four core technologies: cell sources, culture media, scaffolds, and bioreactors (Figure 1). Current methods for sourcing cells rely heavily on harvesting stem cells from fresh meat, a resource‐intensive and commercially unviable process (Swarup et al. 2021). Similarly, although animal‐derived serums and scaffolds are effective, concerns regarding animal welfare and high costs have been raised (Seah et al. 2021; Tyagi and Mani 2023). Additionally, the current operational capacity of bioreactors is limited to millimeter‐scale, posing significant barriers to mass production (Norris et al. 2022). It is, therefore, essential to overcome these challenges if CM is to be transformed into a commercially viable industry.

Media optimization has been identified as a critical factor in lowering production costs, as summarized in several reviews (Flaibam et al. 2024; Gomez Romero and Boyle 2023; Olenic and Thorrez 2023). Yet, there is a paucity of comprehensive reviews that assess the potential of four critical technologies (cell line development, media optimization, scaffold fabrication, and bioreactor design) both individually and within an integrated framework to improve cost efficiency and scalability. This article aims to offer an in‐depth analysis of recent advancements and propose a systematic, integrated approach for future developments with a focus on CM cost‐effective, large‐scale production. It also provides a concise overview of the development history, current research focus, and existing products in the CM industry. The ultimate objective of this review is to identify pathways for producing safe, nutritious, and affordable CM at scale, thereby contributing to global food security.

## 2 History and Current Status of Research on CM

**FIGURE 2:**
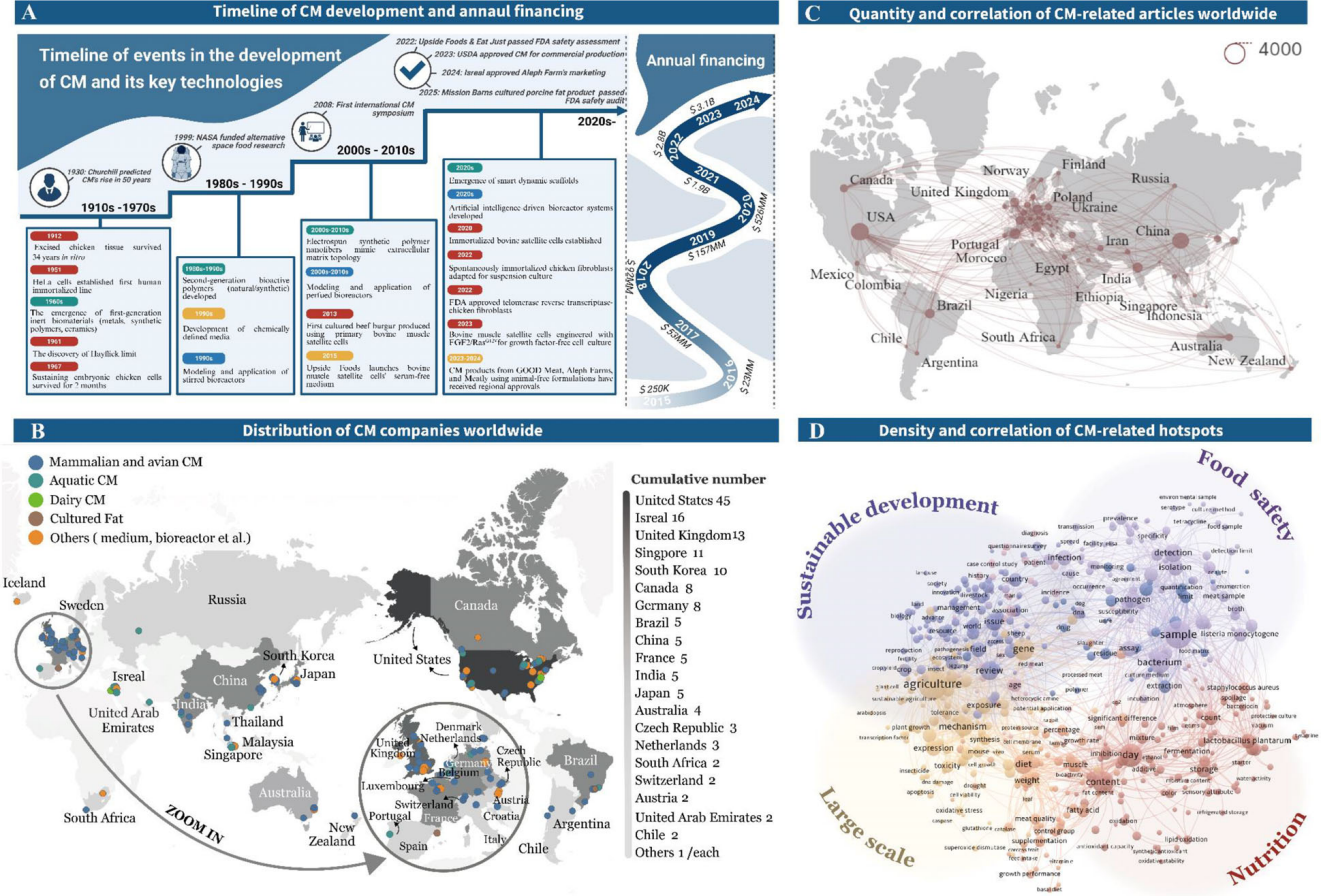
Technological evolution and global landscape of cultured meat (CM). (A) Left: Historical milestones in CM development (1910s–2020s), highlighting critical innovations across four technological domains: cell line engineering (red), scaffold design (green), culture medium optimization (yellow), and bioreactor systems (blue). The development of CM is shown above the timeline; right: annual global CM financing trends (2015–2023); (B) geographical distribution of CM enterprises categorized by operational specialization; (C) quantity and correlation of CM‐related articles in various countries, with the circles in the legend representing 4000 articles; (D) the density and relevance of CM‐related hotspots in the current published studies, mainly in the four areas of sustainable development, food safety, mass, and nutrition. *Source*: (A) Good Food Institute, (B) PitchBook, (C) Web of Science, (D) Web of Science.

The timeline of CM development is illustrated in Figure 2A. CM traces its origins to Alexis Carrel's experiment demonstrating sustained viability of chicken embryonic tissues through continual nutrient replenishment—an early milestone for in vitro tissue engineering (Carrel 1912). Although cell line innovation began with the establishment of immortalized HeLa cells in 1951, applications in cellular agriculture remained limited until NASA's 1999 project to engineer muscle proteins for astronaut nutrition catalyzed the fusion of tissue engineering and food science (Van Eelen et al. 1999). A pivotal breakthrough occurred in 2013 when Mark Post synthesized the first CM burger using bovine myoblasts cultivated in perfusion bioreactors (PBs) (Woll and Böhm 2018). By 2015, Memphis Meat (now Upside Foods) introduced serum‐free medium (SFM) tailored for bovine muscle satellite cells (MuSCs), eliminating dependency on fetal bovine serum (FBS) (https://upsidefoods.com/). Meanwhile, scaffold technology transitioned from inert polymers to nanofibers (2010s) and dynamic scaffolds (2020s) that respond to stimulus response or biomimetic structures (Qu et al. 2019; Stratton et al. 2016), whereas bioreactor design progressed from static cultures to artificial intelligence‐controlled perfusion systems dynamically regulating pH, oxygen, and shear stress for high‐density cell expansion (Dan et al. 2025). Regulatory frameworks matured in parallel: governments in Singapore (2020), the United States of America (2023), and Israel (2024) successively legalized CM commercialization (Smith et al. 2022). In the United States, the Food and Drug Administration (FDA) and the Department of Agriculture Food Safety and Inspection Service (USDA‐FSIS) formed a dual regulatory framework, facilitating the approval of CM production and marketing (Failla et al. 2023). The European Union classifies CM as a novel food under regulation (EU) 2015/2283, requiring risk assessment by European Food Safety Authority and authorization from the European Commission (Broucke et al. 2023). In China, government initiatives such as the 14^th^ Five‐Year Plans are actively promoting CM research. Conversely, the regulatory framework governing market approval remains underdeveloped, reflecting a cautious approach to commercialization. These regulatory advancements have catalyzed global interest in CM, leading to significant investments (Yun et al. 2024). The Good Food Institute (https://gfi.org/) reported that investment increased significantly before 2020, with annual funding surpassing $3.1 billion by 2023 (Figure 2A), as companies such as Aleph Farms and Mosa Meat obtained financing for pilot‐scale smart bioreactors with a production capacity of 10,000 L. By December 2024, over 150 CM companies operated worldwide, according to data from PitchBook, concentrated in Europe, North America, and Asia (Figure 2B), collectively advancing scalable production through integrated breakthroughs.

By 2024, data from Web of Science indicates that the United States (13.62%), China (11.75%), India (4.29%), Germany (4.26%), Italy (4.23%), and Brazil (4.12%) are the key contributors to CM research (Figure 2C). The influence of government policy in supporting research and funding has been a factor in the publication outcomes. A correlation analysis of published literature reveals four key focus areas driving CM research (Figure 2D). The primary focus is on the potential of CM to address resource management, environmental impact, and animal welfare, aligning with Sustainable Development Goals (SDGs). The emphasis on efficient resource use and reduced environmental impact in CM production is in alignment with SDG 6 (Clean Water and Sanitation), SDG 7 (Affordable and Clean Energy), SDG 12 (Responsible Consumption and Production), and SDG 13 (Climate Action) (Post et al. 2020). Socially, CM has the potential to improve access to high‐quality protein, thereby addressing SDG 1 (No Poverty) in developing countries and SDG 10 (Reduced Inequalities) in developed nations (Nobre 2022). Second, research into CM production technology, encompassing material selection, cell culture, cultivation techniques, and yield optimization, represents a crucial research area. This field of research is driven by the objective of transitioning from small‐scale laboratory production to large‐scale commercial output through optimization of the CM production process (Chen et al. 2022). Third, the sensory attributes of CM constitute another significant research focus. Researchers are concentrating on enhancing the texture and flavor of CM to make it more comparable to traditional meat while potentially offering additional health benefits (Ng et al. 2021). For example, Liu et al. (2022) developed cultured pork fat with a higher level of *ω*‐3 fatty acids than conventional pork, potentially reducing the risks of chronic diseases like cancer and cardiovascular conditions through dietary fat optimization (Ruxton and Gordon 2024). Finally, ensuring the safety of CM is imperative, necessitating comprehensive risk assessments at every stage of the production process. Key risks include zoonotic disease transmission, microbial contamination, physico‐chemical changes, and chemical residues during cell selection and production (Jairath et al. 2021). Other concerns include genetic drift, allergenic scaffolds, and cryoprotectant carryover (World Health Organization 2023). Addressing these risks across all production stages is paramount for the safety and quality of CM products (Lanzoni et al. 2024).

## 3 The Production of Various Species of CM for a More Natural Fit

**FIGURE 3:**
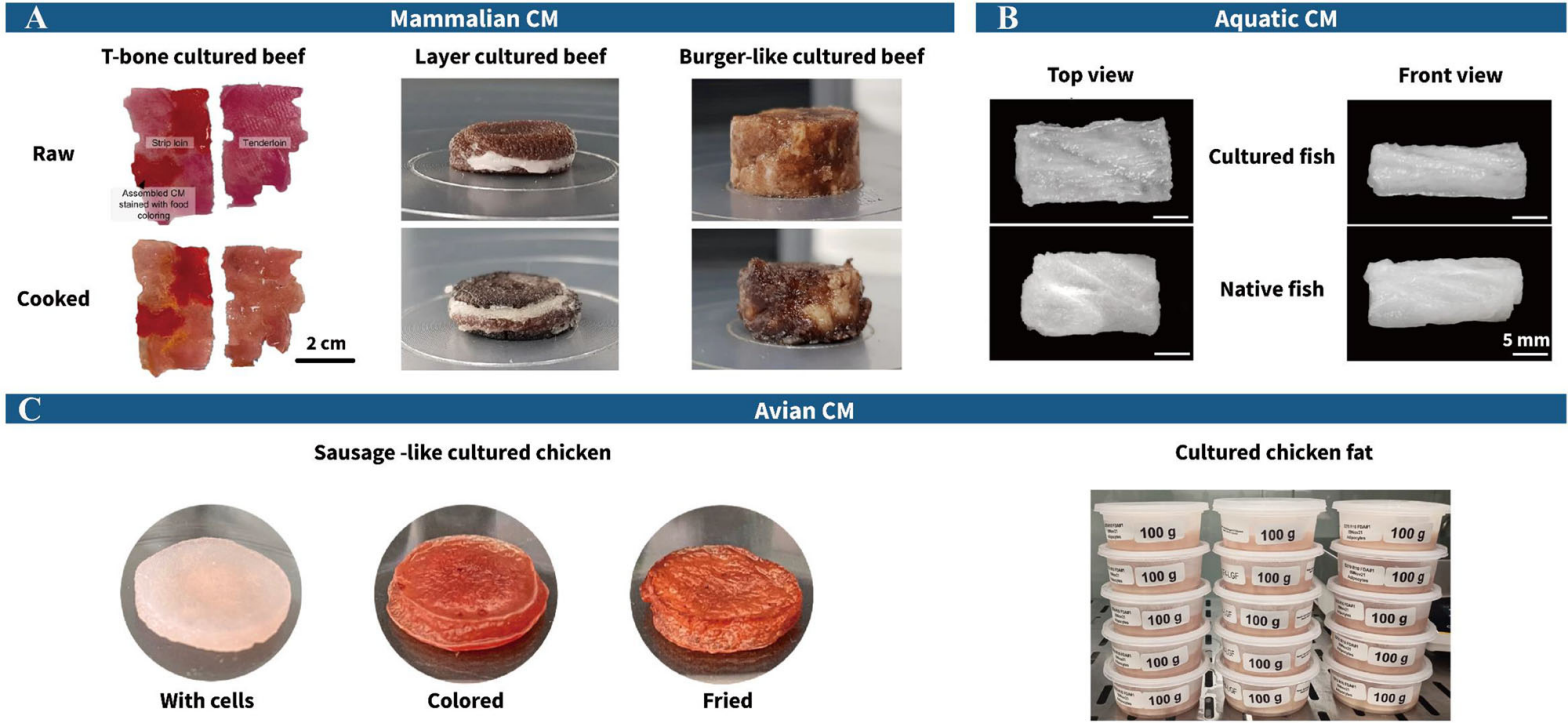
Current cultured meat (CM) products in production. (A) Mammalian CM: T‐bone cultured beef (Lee, Park et al. 2024), layer cultured beef (Yen et al. 2023), and burger‐like cultured beef (Yen et al. 2023); (B) aquatic CM: cultured fish (Xu et al. 2023); and (C) avian CM: cultured chicken (Chen et al. 2023b) and cultured chicken fat (Pasitka et al. 2023).

CM has emerged as a niche product currently manufactured by a limited number of companies on a small scale. Updated data from Yun et al. (2024) indicate CM production efforts prioritize cultured beef (57.35%), pork (46.32%), chicken (44.11%), seafood (34.56%), duck (33.82%), lamb (33.82%), and fat (2.94%). These numbers reflect consumer acceptance rates across meat types, with poultry (89.5%), beef (86.5%), pork/bacon/ham (81.7%), and fish (80.3%) ranking highest, whereas exotic categories such as dog and cat meat receive minimal interest (0.1%) (Wilks and Phillips 2017). Research has begun to yield tangible results in various CM categories, as shown in Figure 3.

### 3.1 Mammalian CM

Mammalian CM (Figure 3A), particularly beef and pork, has garnered significant attention due to the environmental and economic burdens of traditional livestock farming, including high greenhouse gas emissions, substantial water usage, and inefficient feed conversion ratios (Henchion et al. 2021; Specht and Scientist 2020; Warner 2019). The landmark introduction of the first cultured beef hamburger in 2013 marked a turning point (Kang et al. 2021), followed by technological leaps in scaffold innovation, adipose tissue engineering, and muscle‐fat co‐culture system development. Current strategies employ bovine MuSCs, embryonic stem cells (ESCs), and adipose‐derived stem cells (ADSCs) integrated with 3D bioprinting to recreate hierarchical muscle‐fat‐tendon architectures (Ben‐Arye and Levenberg 2019; Furuhashi et al. 2021; Liu et al. 2022). For instance, dynamically cultured bovine ADSC‐derived adipospheres (200–400 µm diameter) serve as modular units for constructing marbled fat‐muscle complexes via extrusion bioprinting, elevating monounsaturated fatty acid content to 47.3% through optimized differentiation protocols (Klatt et al. 2024). Tendon gelatin (gel) bioprinting has facilitated heterogeneous cultured beef with 42 muscle fibers, 28 adipose tissues, and 2 endothelialized vascular bundles, achieving marbling via interfacial protein‐fat emulsification (Louis et al. 2023; Kang et al. 2021). The adipose components enhance textural parameters like consistency, elasticity, and mouthfeel by facilitating muscle protein gelation and forming uniform gel networks through fiber interactions (Fraeye et al. 2020). Such bioengineered structures generate conventional beef‐like flavors during thermal processing via Maillard reactions on polysaccharide–protein scaffolds (Lee, Park et al. 2022), further enhanced by thermo‐responsive flavor compounds embedded in scaffold that release pyrazine/furan volatiles during cooking to mimic natural beef aromas (Lee, Choi et al. 2024). Emerging scaffold innovations also address texture replication. Scaffolds based on chitosan–sodium alginate (SA)–quercetin demonstrate exceptional porosity and mechanical resilience that drive myoblast proliferation and myotube fusion, yielding fried products with tenderness exceeding traditional beef, with additional anthocyanins contained in scaffolds imparting meat‐like coloration and antioxidant functions (Wang et al. 2025). Despite textural parity with conventional meat, cultured beef shows reduced cohesiveness post‐heating (Yen et al. 2023; Table 1), potentially benefiting consumers with swallowing difficulties.

**TABLE 1:** Texture profile analysis of different type cultured meat (CM).

Type	Scaffold	Materials	Hardness	Adhesiveness	Springiness	Cohesiveness	Resilience	Gumminess	Chewiness	References
Cultured beef	Plant‐based scaffold	SA		Undetectable	Maintains high both before and after cooking	Cooked CM is similar to cooked beef	Cooked CM is similar to raw and cooked beef			Lee, Kim et al. (2024)
	Hybrid scaffold	Fish gel and SA	Far lower than beef		Higher compared to beef	Similar to beef			Lower than beef	Lee, Park et al. (2024)
		PC, dialdehyde chitosan, collagen	All groups below beef		Similar to beef after differentiation in all groups			All groups below beef	Higher compared to the blank scaffold	Wang, Zhong, Munawar, Zan et al. (2024)
		PC, dialdehyde chitosan, and collagen	Similar to beef after differentiation		Higher compared to beef			Similar to beef after differentiation	Similar to traditional beef after differentiation	Wang, Zhong, Munawar, Wang et al. (2024)
		Chitosan and collagen	Similar to beef before cooking	Higher than beef patties	Similar to beef before and after cooking	Layer CM is similar to beef patties			CM does not rise after cooking, unlike beef patties	Yen et al. (2023)
	Scaffold Free			Stable over time and with heating, unlike beef	Slightly dropped over time, stable post‐boiling	Similar to beef after boiling			Longer culture made cell sheets chewier, similar to beef	Tanaka et al. (2022)
Cultured pork	Plant‐based scaffold	TVP	Softer than commercial types both raw and fried		Similar to commercial types using PM and SFM				Raw CM with PM, fried CM with SFM similar to commercialized type	Guan et al. (2023)
		PWP	Rising after differentiation but still lower than pork		Twice as much pork			Higher after induction	Rising to similar to pork after differentiation	Zheng, Chen et al. (2022)
		Soybean protein, wheat protein, and peanut protein	Higher at 5 × 10^6^ cell density		Different cell densities were smaller than empty scaffolds			Higher at a cell seeding density of 5 × 10^6^	Increased at 5 × 10^6^ cell density	Zheng, Shi et al. (2022)
	Animal‐based scaffold	Rat tail collagen Type I	Highest for D3^a^, decreases significantly by D6, lowest at D9		Lowest at D6, higher at D3 and D9	Highest at D3, diminishes at D6, similar at D9			Highest at D3, decreases at D6, lowest at D9	Zheng et al. (2021)
	Hybrid scaffold	SA and PVA	Similarly, in conventional pork and CM		Similarly, in conventional pork and CM	Similarly, in conventional pork and CM			Similarly, in conventional pork and CM	Ding et al. (2023)
Cultured chicken	Hybrid scaffold	GG and Type A gel	Scaffolds with cells rising to resemble chicken				Scaffolds with cells rising to resemble chicken	Scaffolds with cells rising to resemble chicken	Scaffolds with cells rising to resemble chicken	Chen et al. (2023b)
Cultured fish	Hybrid scaffold	Porcine and fish gels, hyaluronic acid, silk fibroin, and chitosan	Similarly, in fish and CM		Similarly, in fish and CM		Similarly, in fish and CM	Similarly, in fish and CM	Lower chewiness compared to fish	Xu et al. (2023)

Abbreviations: CM, cultured meat; gel, gelatin; GG, gellan gum; PC, proanthocyanidins; PM, proliferation medium; PVA, polyvinyl alcohol; PWP, peanut wire‐drawing protein; SA, sodium alginate; SFM, serum‐free media; TVP, textured vegetable protein.

^a^ D refers to day in the sentence.

For cultured pork, development focuses on multidimensional flavor simulation, nutritional enhancement, and texture regulation. Gas chromatography–mass spectrometry reveals volatile organic compound profiles highly comparable to conventional pork, though benzaldehyde levels need adjustment (Lee, Park et al. 2024; Song, Liu, Zheng et al. 2022; Supporting Information 1). The PK15H cell line, cultured with 5–10 mM bacterial heme, achieves pork‐like pigmentation and flavor while reducing medium costs, expressing cytochrome P450 and glutathione peroxidase stably (Seo et al. 2024). Despite higher moisture and lower protein/fat concentrations compared to conventional products (Olenic and Thorrez 2023), polysaccharide–protein hydrogel‐based cultured pork meets essential amino acid index standards for commercial pork tenderloin (Guan et al. 2023; Supporting Information 2). Achieving macronutrient and micronutrient balance remains challenging (Tanaka et al. 2022). Recent work by Jeong et al. (2022) dynamically regulated muscle‐to‐adipocyte ratios (9:1 to 7:3), leveraging insulin‐like growth factor‐1 (IGF‐1) secretion to promote adipogenesis and prevent nutrient competition, achieving conventional meat‐like cooking properties (Palm and Thompson 2017). Sorghum prolamin‐based 3D porous scaffolds (PSs) guide aligned co‐culture of porcine MuSCs and ADSCs, yielding cultured pork with 22.9% protein content and enhanced sensory attributes through natural anthocyanin incorporation (Su et al. 2024). The integration of topographical guidance with electrostimulation techniques may present cost‐effective solutions for texture modulation in scaled production (elaborated in Section 4.3.4). Future research must optimize scaffold materials for industrial production, evaluate cooking qualities against conventional meat, and prioritize cost reduction while maintaining sensory authenticity to improve consumer acceptability.

### 3.2 Aquatic CM

Aquatic CM development is motivated by environmental concerns, including Japan's nuclear wastewater discharge impacting marine ecosystems, alongside overfishing and antibiotic overuse in aquaculture (Xia et al. 2024). Fish cells offer intrinsic advantages like hypoxia tolerance, pH adaptability, genetic stability, slow aging, and high proliferation capacity, making aquatic CM promising (Rubio et al. 2019; Fan et al. 2017; Kim et al. 2018). However, high production costs remain a critical bottleneck. Recent advancements in low‐cost scaffold materials and optimized culture strategies have reduced expenses significantly. Tsuruwaka and Shimada (2022) developed bioinks using fibroblast‐like cells from discarded fish fins, whereas Dong et al. (2024) enhanced the proliferation efficiency of *Carassius auratus* muscle cells by incorporating *Auxenochlorella pyrenoidosa* protein extract (APE) in low‐serum media. Agricultural byproduct utilization, such as rice bran‐gel composite scaffolds, improved elastic modulus by 1.8‐fold, delayed hydrogel degradation, reduced costs to 37% of pure gel systems, and promoted piscine satellite cell proliferation and differentiation (Xin et al. 2024). To replicate seafood's nutritional matrix, Xu et al. (2023) achieved compositional parity with native tissue (60.2% muscle and 39.8% fat) through 3D bioprinting of yellowtail‐derived cells and fish gel. Plant‐derived glucosyl nanoparticle‐embedded gel/SA hydrogel scaffolds enhanced muscle fiber density and differentiation efficiency through nanotopography‐induced integrin–cytoskeleton mechanotransduction, attaining tissue characteristics comparable to natural fish after 15 days (Niu et al. 2025). Although textural fidelity approaches conventional benchmarks, quantifiable discrepancies persist—notably reduced chewiness compared to traditional fish (Table 1), potentially attributable to water redistribution within muscle fibers. Emerging solutions such as algae‐based scaffolds simultaneously supported tissue maturation and incorporated umami‐enhancing nucleotides to mimic natural flavor profiles (Ben‐Arye and Levenberg 2019). Future research aims to fortify CM with *ω*‐3 fatty acids, vitamins, antioxidants, and bioactive peptides for enhanced nutritional value and market competitiveness. With global corporations increasingly investing in aquatic CM (Figure 2B), interdisciplinary efforts in biotechnology, food science, and consumer research are crucial for overcoming challenges and accelerating commercial adoption.

### 3.3 Avian CM

Poultry muscle, recognized for its homogeneous texture, slender fiber architecture, and minimal intramuscular fat, offers inherent advantages for CM production compared to complex mammalian or seafood tissues, theoretically simplifying biomass assembly. Recent advancements include Chen et al. (2023b) generating structured cultured chicken by inoculating chicken MuSCs onto Ca^2+^ crosslinked gellan gum‐gel scaffolds, achieving texture profile analysis results (hardness, gumminess, resilience, chewiness) comparable to conventional chicken (Table 1). Ma et al. (2024) improved resemblance by sequentially exposing chicken fibroblasts to myogenic and adipogenic stimuli, controlling intramuscular lipid deposition to match natural poultry while maintaining protein alignment for textural fidelity. Commercial viability has been further underscored by Eat Just Inc., which secured groundbreaking approvals from both the Singapore Food Agency and USDA‐FSIS for its serum‐free cultured chicken products, representing the first regulatory endorsement of its kind globally (Ng et al. 2021). Nonetheless, persistent bottlenecks in avian CM development underscore unresolved challenges. Current immortalization protocols primarily enable fibroblast‐based chicken fat production, as Pasitka et al. (2023) demonstrated by integrating immortalized chicken fibroblasts with soy protein scaffolds for cost‐effective cultured chicken. This reliance on non‐myogenic lineages sacrifices microstructural fidelity, particularly in texture replication, where poultry's delicate, short muscle fibers demand ultra‐fine architecture unmet by fibroblast‐driven systems (Zhang et al. 2024). Economic pressures are significant, with conventional chicken retailing at $2.8–3.5/kg, necessitating energy‐intensive bioprocesses while engineering microscale textural complexity. Transitional hybrid strategies, combining minimal avian cell content with cost‐effective plant‐based scaffolds, may bridge the gap until full cellular agriculture achieves technical and economic parity.

## 4 Four Key Techniques for CM Production

### 4.1 Cell Source Selection and Development

The selection and optimization of cell lines is the foundation of CM production, as it directly influences both biological performance and overall production economics. An ideal cell line should exhibit robust growth, efficient differentiation, and compatibility with large‐scale manufacturing processes, while minimizing the dependence on expensive materials such as GFs and serum (Martins et al. 2024). Furthermore, long‐term stability and scalability are essential for sustained industrial application (Albrecht et al. 2024). To achieve these goals, researchers have undertaken extensive exploration and improvement in areas such as cell isolation and purification, cell type selection, and cell engineering. The following sections systematically discuss approaches to optimizing CM cell sources, highlighting key technical aspects, current challenges, and future directions.

#### 4.1.1 Separation of Cells

The acquisition of high‐quality source cells is a critical prerequisite for CM production, as it lays the groundwork for efficient expansion and differentiation in subsequent stages of the bioprocess. Small tissue samples are collected through needle biopsies or surgical incisions, after which the muscle tissue is minced and enzymatically digested with agents like collagenase II (Ben‐Arye and Levenberg 2019). Alternatively, explant techniques can be employed. However, these processes are both costly and yield relatively low quantities of cells. For example, collagenase digestion costs approximately $500–$2000/g of tissue (sampling 0.5 g at a time) and yields approximately 4 × 10^4^ cells/g (Melzener et al. 2021; Post et al. 2020). Given these challenges, the development of efficient cell separation and purification techniques is paramount to maximize yields and reduce costs.

There are currently three main approaches to cell separation and purification. The first one is density gradient centrifugation, which separates cells based on their density and is considered an economical option requiring minimal equipment. Miersch et al. (2018) employed Percoll gradients ranging from 25% to 70% to isolate MuSCs accumulating between the 40% and 50% density interfaces. After 8 days in culture, about 95% of them expressed *MyoD*. Upon differentiation, the cells achieved a fusion rate of around 25%. Notably, about 62.6% ± 26.5% of the resulting myotubes were medium to large in size, exceeding 100 µm. Nevertheless, there are limitations to this method, including its low throughput, its susceptibility to contamination by cells of similar density, and its inability to handle large samples efficiently (Choi et al. 2021). The second approach involves magnetic‐activated cell sorting and fluorescence‐activated cell sorting (FACS), which use specific markers to achieve high‐purity cell sorting. Although these methods deliver exceptional precision, they are hindered by high operational costs, cross‐contamination risks, bioaerosol pollution, and inefficiencies in processing large volumes (De Wijs et al. 2017). To address these issues, researchers have combined FACS with microfluidic technology, achieving remarkable enrichment of breast cancer cells from 0.6% to 91%, but the technique's low throughput still limits its ability to process large volumes of cells quickly (Cai et al. 2020). The pre‐plating method, on the other hand, is simpler, more cost‐effective, and better suited for mass production (Rasmussen et al. 2024). Guan et al. (2022) demonstrated that pre‐plating for just 0.5 h, followed by 20 days of expansion, yielded (2.19 ± 0.16) × 10^8^ MuSCs—five times higher than conventional FACS methods (Li, Wang et al. 2022). This method can be further optimized by re‐plating and incubating for five minutes to reduce interference from non‐target cells. The obtained MuSCs exhibit nearly 100% expression of *PAX7* and *MyoD* (Yoshioka et al. 2020). This method often suffers from long cycle time and poor reproducibility. Besides, competition from non‐target cells during culture can lower the overall purification efficiency (Pasut et al. 2012).

As cell separation is the foundational step in CM production, future research should focus on developing a regulated, efficient, and scalable process. A combined approach leveraging the strengths of different techniques is essential. For instance, automated pre‐plating systems can enhance the initial enrichment of target cells. Density gradient centrifugation can then be optimized to selectively remove non‐target cells by refining the gradient medium and range for improved efficiency. Finally, isolated cells from each batch should be validated using FACS technology to ensure high accuracy and purity. This integrated strategy will significantly improve the scalability and cost‐effectiveness of CM production.

#### 4.1.2 Commonly Used Cell Lines

**FIGURE 4:**
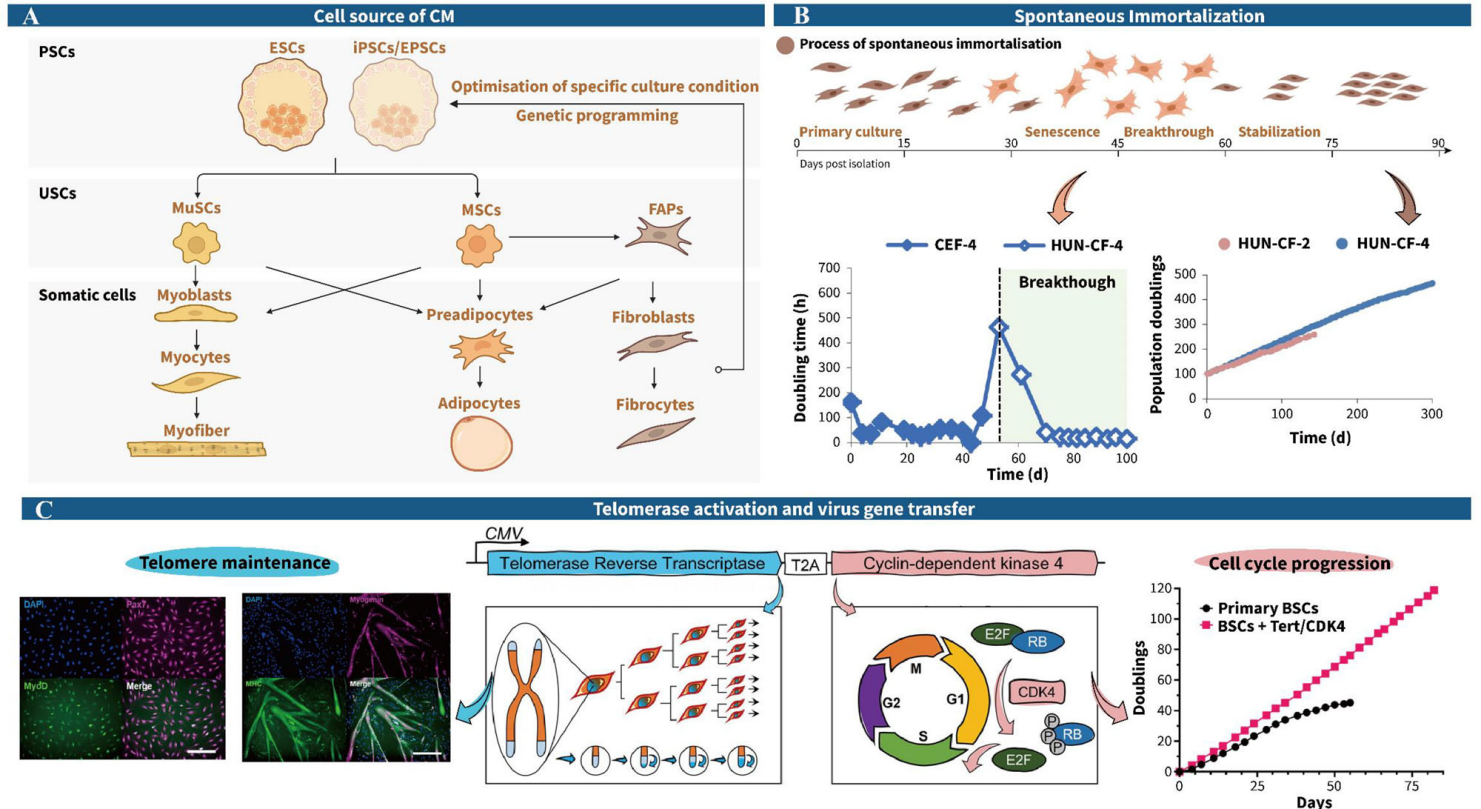
Cell sources and construction of cell lines for cultured meat (CM). (A) Cellular sources commonly used for CM and their differentiation processes. Pluripotent stem cells (PSCs), including embryonic stem cells (ESCs), induced pluripotent stem cells (iPSCs), and expanded potential stem cells (EPSCs), can be induced to generate unipotent stem cells (USCs) such as muscle stem cells (MuSCs), mesenchymal stem cells (MSCs), and fibro‐adipogenic progenitors (FAPs). These USCs further differentiate into somatic cell types, involving myoblasts, adipocytes, and fibrocytes, enabling the generation of structured and functional cultured meat products through coordinated cellular maturation and tissue organization; (B) primary chicken fibroblasts (CEF‐4) experienced a breakthrough around Day 50, forming an immortalized cell line (HUN‐CF‐4) within 10–30 days of subsequent senescence. These cells demonstrated stable growth over 466 population doublings (Pasitka et al. 2023); (C) the CMV promoter drives the expression of telomerase reverse transcriptase (TERT) and cyclin‐dependent kinase 4 (CDK4) genes, facilitating cell expansion and long‐term maintenance. The modified bovine muscle stem cells (MuSCs) retained their ability to differentiate and were capable of sustaining over 120 passages (Stout, Arnett et al. 2023).

Current research focuses on three main categories of cell lines: pluripotent stem cells (PSCs), unipotent stem cells (USCs), and somatic cells (Figure 4A). Each type has distinct advantages and limitations in addressing the challenges of large‐scale and cost‐effective CM production.

##### 4.1.2.1 Pluripotent Stem Cells

PSCs hold great promise for CM production on account of their high self‐renewal capacity and potential to establish cell banks (Jara et al. 2023). Currently, ESCs and induced PSCs (iPSCs) have established harvesting practices in mammals (Bogliotti et al. 2018; Gao et al. 2019; Su et al. 2021; Vilarino et al. 2020), poultry (Kim et al. 2017; Xiong et al. 2020), and aquatic species (Xu et al. 2021; Yi et al. 2010) (Supporting Information 3). However, their reliance on GFs and feeder layers to maintain stability and pluripotency results in high production costs and ethical concerns. For example, maintaining the stability and pluripotency of bovine and ovine ESCs requires fibroblast GF‐2 (FGF2) and WNT signaling inhibitors, which support karyotype stability and pluripotency for up to 70 and 40 passages, respectively (Bogliotti et al. 2018). Rabbit ESCs cannot colonize without feeder layers and require a feeder cell density of 6 × 10^3^/cm^2^ to maintain their proliferation and pluripotency (Honda et al. 2008). Efforts to replace feeder layers with alternatives such as B27 or small molecule inhibitors have shown potential but require long culture periods (taking up to 37 days) (Zhu, Gao et al. 2023) or exhibit halved proliferation rates (Soto et al. 2021). Similarly, iPSCs face challenges, including the need for specific reprogramming factors like lysine‐specific demethylase 4A, estrogen‐related receptor β, and stage‐specific embryonic antigen‐4, which raise safety concerns in CM production (Botigelli et al. 2022; Li et al. 2011; Su et al. 2021). Following this, researchers are increasingly exploring expanded potential stem cells (EPSCs) due to their strong proliferative capacity, genomic stability, and feeder‐free growth. Porcine and bovine fibroblasts reprogrammed into PSCs using doxycycline and eight exogenous factors have remained undifferentiated for over 20 and 63 passages, respectively (Gao et al. 2019; Zhao et al. 2021). Still, the dependence of these cells on doxycycline and Mek1/2 inhibitors raises concerns about drug residues, stringent culture conditions, and the high cost of CM production.

Among farm animals, pigs, cattle, sheep, rabbits, and fish represent the most established models for PSC derivation, with validated ESC lines and iPSC systems (Botigelli et al. 2023). Future research should focus on standardizing the isolation, culture, and lineage establishment of ESCs across multiple species. This would reduce the need for animal embryos and further the understanding of early embryonic pluripotency regulation (Chen, Tang et al. 2024; Gao et al. 2019; Honda et al. 2008; Vilarino et al. 2020). Additionally, optimizing reprogramming strategies and exploring natural alternatives to synthetic drugs in generating EPSCs could help lower costs and mitigate adverse effects. Nonetheless, inducing PSCs to differentiate into USCs and further into somatic cells remains complex and costly. For large‐scale, cost‐effective CM production, these disadvantages outweigh the benefits of PSC self‐renewal. As a result, most research continues to focus on the use of USCs and somatic cells.

##### 4.1.2.2 Unipotent Stem Cells

USCs are a practical choice for CM production given their abundance in mature tissue, ease of isolation, and ability to differentiate into specific cell types (Xu et al. 2023; Yen et al. 2023; Zernov et al. 2022). MuSCs are commonly used to produce muscle tissue because they closely mimic natural muscle and have multipotency. However, maintaining their long‐term proliferation necessitates repeated supplementation of GFs (Supporting Information 3). These factors exhibit diminishing efficacy beyond around 10 passages, thereby escalating process complexity and costs. In some cases, the differentiation of MuSCs from chicken and fish requires additional components such as horse serum and embryo extracts, which not only raise ethical concerns but also increase production costs due to the reliance on fertilized eggs (Dong et al. 2024; Hong and Do 2024). Fat content is another critical factor in replicating the texture and flavor of conventional meat. Although mesenchymal stem cells (MSCs) have been used for adipocyte differentiation, their reliance on costly inhibitors and limited fat yield (0.137% compared to 6.7% in traditional beef) highlight their inefficiency (Lee, Park et al. 2024). Some studies suggested that this shortfall could be addressed by adding exogenous fatty acids, such as oleic acid and palmitoleic acid, which have been shown to nearly triple the lipid reserves in differentiated MSCs (Louis et al. 2023). Moreover, they found that fibro/adipogenic progenitors demonstrated superior adipogenic differentiation in 3D culture, with a 4.5‐fold increase in the white adipose‐related gene *PPARγ* compared to 2D culture. This approach further increased CM's fat content to approximately 22%. A potentially more efficient approach may be to combine oleic acid with microRNA‐100 to induce intramuscular fat deposition in MuSCs, which could streamline the process by avoiding the need to culture two different cell types, thus reducing the reliance on costly GFs (Mir et al. 2022).

USCs’ differentiation relies heavily on GFs as well, and the extracted cells are disposable, making them dependent on mature cell extraction techniques and standardized culture conditions. Future research is expected to establish comprehensive cell banks that will allow more efficient use of various cell types, reducing the need for ongoing extractions and providing a more sustainable basis for large‐scale CM production.

##### 4.1.2.3 Somatic Cells

Somatic cells, particularly myoblasts, are considered a promising cell source for CM because of their rapid proliferation, high differentiation efficiency, and ability to generate muscle tissue similar to natural muscle (Supporting Information 3) (Kong et al. 2023; Lee, Park et al. 2024). Beyond myoblasts, other somatic cells, such as fibroblasts (Zernov et al. 2022) and pre‐adipocytes (Kawecki et al. 2023), are increasingly used to enhance the texture and flavor of CM by contributing to connective tissue and fat production. Among these, pre‐adipocytes stand out for their superior differentiation efficiency, stability, and controllability compared to MSCs, making them an excellent option for enhancing fat content in CM. For example, Gu et al. (2024) achieved up to 15% fat content by seeding pre‐adipocytes in a composite hydrogel, with lipid droplet sizes ranging from 1500 to 4000 µm^2^. Compared to stem cells, somatic cells are easier to isolate, require simpler culture conditions, and can be directly sourced from adult animals, reducing ethical concerns (Wang, Zhong, Munawar, Zan et al. 2024). Nonetheless, the limited lifespan of primary somatic cells poses a significant challenge for mass production, as they tend to undergo senescence after a finite number of passages. Recent research has explored cell engineering to improve longevity and scalability for more efficient CM production.

#### 4.1.3 Cell Immortalization Technology

A typical 0.5 g tissue biopsy yields about 1 × 10^4^ cells, but achieving the quantities needed for large‐scale biomanufacturing requires 30–40 population doublings (Post et al. 2020). However, primary cells typically survive limited passages before reaching senescence, severely limiting their industrial viability (Zernov et al. 2022; Zheng, Chen et al. 2022; Zheng, Shi et al. 2022; Zheng et al. 2021). The repeated need to harvest fresh stem cells creates significant logistical and economic barriers, hindering scalability. As a promising alternative, somatic cells combined with immortalization techniques offer a time‐efficient, cost‐effective, welfare‐friendly, and scalable solution. Immortalization involves modifying somatic cells to enable indefinite self‐renewal by introducing telomerase or altering genes that regulate the cell cycle and prevent telomere shortening (De Bardet et al. 2023). Although somatic cell immortalization has notable advantages, the range of commercially available immortalized somatic cells remains limited, with most applications restricted to mouse‐derived C2C12 cells (Chen, Dai et al. 2024; Seo et al. 2023). Therefore, expanding the diversity of immortalized somatic cells and developing scalable production methods are critical areas for future research.

Several methods are available for somatic cell immortalization, including spontaneous immortality, telomerase activation, and virus‐mediated gene transfer. Spontaneous immortality, a non‐genetic modification technique, allows cells to be cultured in vitro until they acquire self‐renewing properties (Soice and Johnston 2021). To illustrate, Pasitka et al. (2023) successfully cultured fibroblast cell lines from Ross 308 chickens and Israeli Baladi chickens, achieving over 259 and 466 passages, respectively, after 30–70 days of repeated division (Figure 4B). Similarly, Krishnan et al. (2023) immortalized *Paralichthys olivaceus* myoblasts that retained their ability to differentiate after just 20 passages. Saad et al. (2023) also achieved spontaneous immortality in mackerel myoblasts, which proliferated every 24.3 h after 37–43 generations. Although these cells demonstrated promising fatty acid composition, their polyunsaturated fatty acid (PUFA) levels were relatively low. This indicates a need for further research, such as incorporating docosahexaenoic acid or its precursors, to enhance PUFA levels and improve the nutritional profile of CM. Although spontaneous immortality has shown potential, it has yet to be commercialized due to challenges such as inconsistency, lack of control, genomic instability, and incomplete safety evaluations. One notable issue is that spontaneous immortalized bovine cells lose their ability to differentiate into myotubes, necessitating the use of gene transfer techniques to introduce and overexpress the *MyoD* gene to restore differentiation capacity (Jin et al. 2006). In contrast, telomerase activation and virus‐mediated gene transfer techniques offer shorter production cycles, better controllability, and greater reproducibility. Stout, Arnett et al. (2023) reported that combining bovine telomerase reverse transcriptase with cyclin‐dependent kinase 4 achieved immortalization of bovine MuSCs, maintaining their differentiation capacity and enabling over 120 passages of long‐term proliferation (Figure 4C). Nonetheless, introducing oncogenes or telomerase genes could pose significant safety concerns. Although the FDA has approved telomerase reverse transcriptase‐immortalized chicken fibroblasts as safe, further studies are required to address long‐term food safety concerns (Bennie et al. 2025). Though somatic cell immortalization techniques are still developing, they offer promising solutions to address the high costs and limited scalability in CM production.

Future scalable solutions may focus on utilizing CRISPR gene‐editing technology to enhance immortalization methods, as its precision, low cost, and efficiency could provide stable and safe cell source for CM production (De Bardet et al. 2023). Although CRISPR enables transient delivery of Cas9 complexes via mRNA or ribonucleoprotein particles (RNPs) to minimize off‐target effects and avoid genomic integration risks, challenges persist. For instance, cell‐type‐dependent editing efficiency and residual off‐target activity, particularly in primary somatic cells, present critical hurdles for industrial‐scale applications (Xu et al. 2024). These limitations could compromise the uniformity and safety of immortalized cell lines. To address this, emerging strategies such as high‐fidelity Cas9 variants (e.g., HypaCas9) and machine learning‐guided sgRNA design are being explored to improve specificity (Abbasi et al. 2025). Additionally, optimizing delivery systems (e.g., lipid nanoparticles or electroporation protocols) tailored to agricultural‐relevant cell types could enhance editing efficiency while reducing variability (Chen, Han, et al. 2024). For example, knockout of cell‐cycle inhibitors (p15/p16) in muscle progenitors using CRISPR‐RNPs has already demonstrated enhanced proliferation without compromising differentiation capacity, suggesting a scalable pathway for CM biomanufacturing (Genovese et al. 2019). Future efforts should prioritize standardized editing protocols, rigorous off‐target screening, and cross‐species validation to ensure both efficacy and regulatory compliance, ultimately advancing CRISPR‐engineered cell lines toward commercial viability.

Conclusively, the selection of CM cell lines depends upon technological readiness, cost‐effectiveness, and the extent of meat simulation. Estimates of culture medium components and market reagent prices indicate that the production cost for PSCs is $10^3^/L, whereas USCs and somatic cells are priced at $10^2^/L, thereby providing somatic cells with a cost advantage. Product segmentation further dictates cellular strategies. Structured CM products (e.g., steak analogs) demand co‐cultured muscle and adipose cells to replicate marbling patterns and fibrous textures, catering to premium markets. In contrast, unstructured formats like burger patties and sausages prioritize cost efficiency through hybrid formulations—blending minimal muscle or fat cell biomass with plant‐based proteins (e.g., pea or legume isolates). This approach slashes cell culture requirements, reducing production costs to be competitive with conventional meat and more accessible for mainstream adoption. As cell immortalization technologies, especially gene editing techniques, continue to advance and become more widespread, they are expected to extend cell lifespan and reduce culture costs, further narrowing the cost gap between structured and non‐structured meat, thus driving the adoption of CM technology in broader markets.

### 4.2 The Construction of SFM

To create a growth environment similar to the body, current cell culture media typically contain organic compounds (including carbon and nitrogen sources), inorganic substances (including salts and vitamins), animal serum, and cytokines (Supporting Information 3). However, the high cost of components such as serum or GFs results in expensive culture media, ranging from $200 to $500/L (Seah et al. 2021; Stout et al. 2022). Approximately 10^14^ cells are required to produce 1 t of CM, requiring 10,000 L of media at a cell density of 10^7^ cells/mL (Chen et al. 2022). To meet the global demand for meat by 2050, when the population is expected to reach 9.8 billion and average meat consumption is estimated at 50 kg/person per year, would require an astonishing 4.9 trillion L of culture media (Sijpestijn et al. 2022). In addition to the cost, the commonly used FBS raises ethical concerns and poses risks related to antibiotic resistance and pathogen contamination (Ong et al. 2021; Chelladurai et al. 2021). Cost reduction and scalable production of CM thus require urgent solutions, including the development of SFM and methods to recycle culture media.

#### 4.2.1 Principles of SFM Construction

**FIGURE 5:**
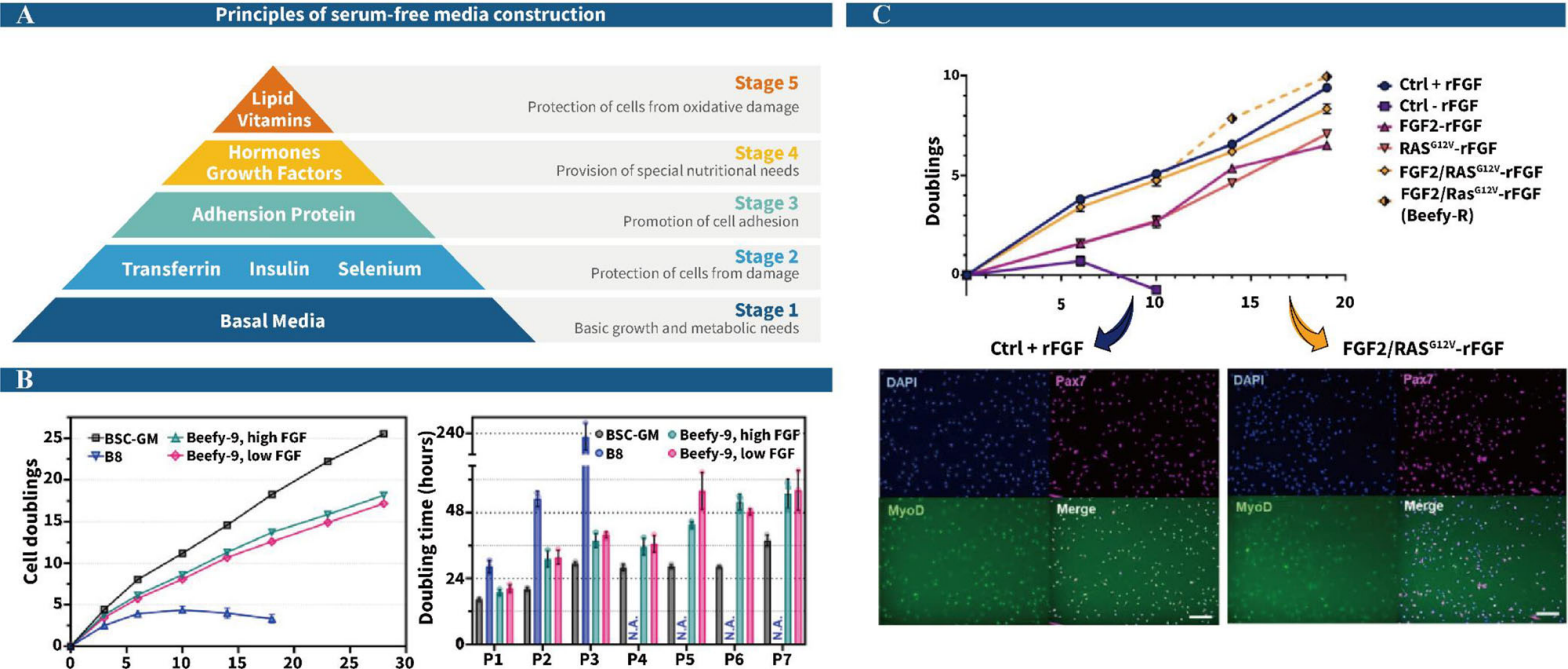
The construction of serum‐free medium (SFM). (A) The five key principles of SFM construction; (B) B8 media with the addition of 800 µg/mL recombinant albumin (Beefy‐9) significantly improved cell growth, increased the number of doubling generations, and shortened the doubling time, although not as effectively as serum (Stout et al. 2022); (C) the growth rate of FGF2/RAS G^12V^‐engineered cells was not significantly different from that of control cells with rFGF in Beefy‐9 and Beefy‐R, and the engineered cells expressed high levels of *Pax7* and *MyoD* genes (Stout, Zhang et al. 2023).

SFM are designed without serum components and are categorized into chemically defined and undefined types (Van der Valk et al. 2010). FBS has traditionally been used as a cell culture supplement because it contains approximately 1800 proteins, as well as various GFs, hormones, amino acids, and vitamins that promote cell adhesion, growth, and activity (Chelladurai et al. 2021; Yao and Asayama 2016). To replicate the function of FBS, SFM must include four key elements (Figure 5A) (Van der Valk et al. 2010). The first essential component is insulin–transferrin–selenium (ITS), which supports cell growth, supplies iron, and prevents oxidative damage (Luck and Mason 2012; Zeng 2009). Second, adhesive proteins must be used to promote growth, as the absence of collagen and matrix gel can hinder cell attachment. Studies show that the application of 1.5 µg/cm^2^ of truncated vitronectin (Vtn‐N) enhances cell adhesion by fourfold compared to untreated controls, effectively supporting growth during passaging (Stout et al. 2022). The third key element is the inclusion of hormones and GFs, which activate vital signaling pathways, regulate metabolism, and prevent apoptosis to ensure cell survival in serum‐free conditions. These GFs can be divided into three main groups: (1) insulin and IGF, which activate the PI3K/AKT pathway, enhancing glucose metabolism and lipid synthesis while inhibiting apoptosis factors like caspase‐9 (Machida and Booth 2004); (2) epidermal GF (EGF), hepatocyte GF, FGF, vascular endothelial GF (VEGF), neuregulin 1, and dexamethasone (DEX), which both activate the PI3K/AKT and RAS‐MAPK/ERK pathways, stimulating cell metabolism and driving cell division and growth (Anderson 2016; Golding et al. 2007). Additionally, EGF and DEX activate the mTOR pathway to promote protein synthesis, thereby increasing muscle cell size and supporting myotube fusion (Wang et al. 2015), whereas VEGF activates the Notch pathway to enhance muscle cell self‐renewal (Verma et al. 2018); (3) transforming GF (TGF)‐β3 and activin A, which activate the TGF‐β signaling pathway to regulate muscle cell differentiation while inhibiting fibroblast proliferation, thus promoting muscle growth and preventing fibrosis (Yin et al. 2020). Lastly, lipids and antioxidants are crucial for maintaining cell membrane stability and minimizing oxidative stress (Doseděl et al. 2; Wallert et al. 2021). Mayengbam et al. (2023) found that adding 10 µg/mL of high‐density lipoprotein cholesterol to colon cancer cell cultures boosted glucose uptake, utilization, and cell proliferation by 2.5 times.

#### 4.2.2 Development and Optimization of SFM

The development of SFMs has enabled cell type‐specific optimization to support cellular growth, proliferation, and differentiation in CM production (Table 2). Although most SFM formulations developed for CM are designed for use with USCs and somatic cells, fewer studies focus on optimizing SFMs for PSCs, primarily due to the inherent limits of extended culture cycles, high costs, and complex procedures. Consequently, current research prioritizes SFM development for the former two.

**TABLE 2:** The formulation of serum‐free medium (SFM).

References	Name	Cell type	Basal media	Alternative(s)								Results
				Growth factor(s)	Hormone(s)	Other protein(s)	Amino acid(s)	Vitamin(s)	Lipid(s) and fatty acid(s)	Mineral(s)	Other(s)	
Kuo et al. (2020)	B8	iPSCs	DMEM/F12	FGF‐2 and TGF‐β1	Insulin	Transferrin	—	Ascorbic acid 2‐phospho‐	—	Sodium selenite	—	iPSC strains were successfully cultured in B8 and maintained pluripotency over 100 passages
Stout et al. (2022)	B8	MuSCs	DMEM/F12	FGF‐2, TGF‐β3, and NRG1	—	Transferrin, rAlbumin	—	2‐Phospho‐l‐ascorbic acid trisodium salt	Linoleic acid and oleic acid	Sodium selenite	—	B8 reduced serum requirements in bovine MuSCs but could not eliminate them completely
	Beefy‐9	MuSCs	DMEM/F12	FGF‐2, TGF‐β3, and NRG1	—	Transferrin, recombinant human albumin, and Vtn‐N	—	2‐Phospho‐l‐ascorbic acid trisodium salt	Linoleic acid and oleic acid	Sodium selenite	—	Beefy‐9 enables sustained cell growth over seven passages, doubling every 39 h
	Beefy‐9 SFDM	MuSCs	DMEM/F12	EGF and IGF‐1	—	—	—	—	—	—	—	After 6 passages, Myhc expression increased, but cell senescence reduced myotube nuclear density
Stout, Zhang et al. (2023)	Beefy‐9^−^FGF	iBSCs	DMEM	TGF‐β3 and NRG1	—	Transferrin, recombinant human albumin, and Vtn‐N	—	2‐Phospho‐L‐ascorbic acid trisodium salt	Linoleic acid and oleic acid	Sodium selenite	—	Cells engineered for FGF2 autocrine signaling grow without needing external FGF
	SFDM	iBSCs	DMEM	IGF‐1 and EGF	—	—	—	—	—	—	—	Engineered cells exhibited half the nuclear fusion rate compared to those treated with FGF
Messmer et al. (2022)	SFB	MuSCs	DMEM/F‐12	EGF‐1	—	Albumin	MEM amino acids	l‐Ascorbic acid 2‐phosphate	—	Sodium Selenite	—	SFB increased nuclei count by 76% over DMEM/F‐12, improving cell survival but not the fusion index
	SFDM	MuSCs	DMEM or DMEM/F‐12	EGF‐1	Insulin	Albumin and transferrin	Amino acid solution	Vitamin C	LPA	Sodium Selenite	—	Myogenic differentiation levels matched those achieved during serum starvation
Skrivergaard et al. (2023)	SFGM	MuSCs	DMEM/F‐12	FGF‐2	Insulin	Fetuin, BSA, and transferrin	—	—	—	Selenium	—	A 32‐fold multiplier in 5 days
Guo et al. (2014)	SFDM‐1	MuSCs	DMEM	EGF	Insulin	BSA	—	—	—	—	—	Although multinucleated myotubes form, continued use of this medium causes them to separate within a few days, hindering further maturation
	SFDM‐2	MuSCs	DMEM	GDNF, BDNF, IGF‐1, CNTF, neurotrophin‐3, and neurotrophin‐4	—	Shh, Vtn‐N, laminin, and agrin	Glutamax	Retinoic acid	—	—	B27, G5, and cAMP	At Day 10, myotubes reach full maturity, appearing as striated bands characteristic of mature muscle fibers
Dong et al. (2024)	—	MuSCs	Leibovitz's L‐15 medium	—	—	—	—	l‐ascorbic acid	—	—	APE	On Day 3, there was no significant difference in cell numbers between the 10% FBS group and other groups, indicating that the substitute can replace 10% FBS for large‐scale cell culture
Dai et al. (2024)	OP13	Myoblasts	DMEM	bFGF	DEX and insulin	BSA‐V, transferrin, and palmitoyl‐3‐peptide‐5	l‐Glutathione	Vitamin C, Vitamin E, folic acid, and biotin	Cholesterol and lipid concentrate	—	Ethanolamine	Cells rapidly formed Myhc‐positive myotubes, and after 6 generations, growth was compatible with conventional media, apoptosis was reduced, and differentiation could be induced

Abbreviations: APE, *Auxenochlorella pyrenoidosa* protein extract; BDNF, brain‐derived neurotrophic factor; BSA, bovine serum albumin; CNTF, ciliary neurotrophic factor; DEX, dexamethasone; EGF, epidermal growth factor; FGF, fibroblast growth factor; GDNF, glial cell line‐derived neurotrophic factor; iBSCs, immortalized bovine satellite cells; IGF, insulin‐like growth factor; LPA, lysophosphatidic acid; Myhc, muscle protein myosin heavy chain; NRG1, neuregulin 1; SFB, serum‐free base; SFDM, serum‐free differentiation media; SFGM, serum‐free growth media; Shh, sonic hedgehog; TGF, transforming growth factor; Vtn‐N, vitronectin.

Muscle and adipose cell lineages require substantial energy and amino acid supply to sustain growth and proliferation, making amino acid‐rich formulations like Dulbecco's modified Eagle's medium (DMEM) a standard choice for CM production. Although basal media such as DMEM are mass‐produced at low cost and minimally impact overall CM pricing, their animal‐derived components remain costly and prone to supply chain instability. Contemporary SFM formulations predominantly depend on recombinant proteins, which constitute up to 95% of media expenses (Pajcin et al. 2022). Optimizing these formulations with cost‐effective and sustainable components is essential for supporting large‐scale CM production. For instance, Skrivergaard et al. (2023) developed an efficient SFM for bovine MuSCs that achieved 32‐fold proliferation in 5 days, but the medium's cost was driven up by fetuin, priced at $166/g. Even at the effective concentration of 600 µg/mL, fetuin alone cost $100/L. Removing fetuin lowered the cost to $16/L, but this formulation could only sustain MuSCs for three passages (Kuo et al. 2020). To achieve seven passages, 800 µg/mL recombinant albumin (Beefy‐9) was added, increasing the cost to around $36/L (Figure 5B) (Stout et al. 2022), underscoring the indispensable yet costly role of specific proteins.

To reduce reliance on costly additives and enable consistent cell expansion for large‐scale production, researchers are pursuing alternative strategies to replace high‐cost components such as GFs (FGF2, IGF1, etc.), transport proteins (albumin, transferrin, etc.), and adhesion factors (vitronectin, fibronectin, etc.). In the first approach, genetic engineering has been employed to create self‐sustaining cell lines capable of endogenously producing essential GFs. Stout, Arnett, et al. (2023) demonstrated that FGF2/Ras^G12V^‐modified bovine MuSCs achieved comparable proliferation in FGF‐free Beefy‐9 medium to cells supplemented with 40 ng/mL FGF, albeit with 50% lower fusion rates (Figure 5C). A complementary study showed that supplementation of SFM with 10 µM lysophosphatidic acid increased fusion indices to 30%–40%, approaching the efficacy of serum‐containing media. However, such formulations only achieve one‐third of traditional media's differentiation gene expression (e.g., *MYH2*), highlighting the necessity for synergistic protein optimization (Cencetti et al. 2014; Messmer et al. 2022).

In parallel, a second strategy focuses on the exogenous production of recombinant proteins. Several host platforms are available, each with unique advantages and limitations: mammalian, microbial, plant‐based, algal, and insect cell systems. Mammalian systems are particularly adept at producing complex glycoproteins that undergo human‐like post‐translational modifications, accounting for 70% of commercial recombinant proteins (O’ Flaherty et al., 2020). Despite this, their high operational costs, extended culture periods (typically 1–2 weeks), and rigorous sterility demands significantly hinder their applicability in CM production, where cost‐effectiveness is crucial (Rozov et al. 2018). In contrast, microbial systems provide a cost‐effective solution with rapid fermentation capabilities and high production yields, albeit limited to proteins that require minimal post‐translational modifications (McKenzie and Abbott 2018). For instance, *Pichia pastoris* can produce 8.86 g/L recombinant serum albumin through 96‐hour methanol induction, achieving >96% purity and 57% recovery rate post‐purification (Zhu et al. 2018). Nevertheless, these systems frequently generate proteins in the form of inclusion bodies, requiring extensive and expensive downstream processing steps such as solubilization, refolding, and chromatography. These processes can constitute up to 80% of the total production costs, primarily due to the need to eliminate host cell contaminants and restore disulfide bonds (Siew and Zhang 2021). Plant‐based platforms, on the other hand, offer the capability to execute eukaryotic modifications, such as *N*‐glycosylation, while providing lower material costs and minimizing the risk of zoonotic contamination (Xu et al. 2025). Although they typically yield lower protein concentrations (0.01–10 mg/L) and necessitate more intricate purification processes, plant‐based systems are particularly appealing for the production of complex glycoproteins (Karki et al. 2021). Illustrative of this is the use of rice suspension cells to produce FGF2 at a concentration of 28 mg/L, which not only results in a 98% reduction in costs compared to mammalian systems but also successfully preserves iPSC pluripotency (Poudel et al. 2019; Lee, Lee et al. 2022). Likewise, tobacco expression systems have demonstrated the ability to generate ferritin heavy chains at 40 mg/kg, with production costs amounting to just 3% of commercial rates and achieving a purity greater than 90% (Knödler et al. 2023). Algal systems harness photosynthesis to achieve sustainable protein secretion, presenting an environmentally friendly option. However, their scalability is challenged, as evidenced by *Chlorella vulgaris*, which accumulates only 0.26–1.42 ng/g of FGF of biomass (Ali et al. 2025; Bolaños‐Martínez et al. 2022). Insect cell platforms are capable of supporting complex protein folding and glycosylation, making them suitable for certain recombinant proteins. However, they are plagued by prohibitive operational costs, primarily due to their extended production cycles, which can last approximately 30 days (Hong et al. 2022; Rozov et al. 2018). The lengthy culture times increase both the time to market and the overall production expenses, making them less favorable for cost‐sensitive applications like CM production. To address the demands of large‐scale CM production, a strategic approach involves combining microbial systems, which are ideal for producing high volumes of unmodified proteins such as insulin, with plant‐based systems, which are better suited for generating functionally modified eukaryotic proteins like FGF2. This hybrid strategy ensures cost‐effective scalability while maintaining the necessary biological efficacy. Furthermore, recent process optimizations have significantly enhanced production yields; for instance, continuous fermentation has increased yeast β‐galactosidase production by 11‐fold (De Brabander et al. 2023). Looking ahead, advancements such as CRISPR‐mediated expression enhancement and the innovation of self‐cleaving affinity tags hold promise for further reducing production costs (Hu et al. 2018; Leonhardt et al. 2023).

Beyond these production platforms, co‐culture systems that utilize multiple cell types provide a straightforward method to supply essential proteins. For instance, rat hepatocytes treated with *C. vulgaris* extract (CVE) secrete IGF2, and supplementing culture media with 10% CVE induces proliferation‐related gene expression in bovine MuSCs comparable to that achieved with 10% FBS (Yamanaka et al. 2023). Although this approach may not match the proliferation efficiency of traditional serum‐based methods, its cost‐effectiveness and improved biosafety make it a valuable complementary strategy when combined with other techniques.

Capitalizing on amino acid profiles and bioactive functions, plant‐based alternatives also offer scalable and cost‐efficient solutions for sustainable CM production at industrial volumes. Stout, Rittenberg et al. (2023) developed an SFM called Beefy‐R, which uses rapeseed protein isolate to replace albumin in Beefy‐9, resulting in a 14‐fold cost reduction and a 20% decrease in the population doubling time of bovine MuSCs compared to the original formulation. Similarly, Dong et al. (2024) demonstrated that MuSCs cultured in a medium with 5% FBS and 1 mg/mL APE maintained stable proliferation over at least four passages, with elevated levels of flavor‐enhancing amino acids like glutamic acid and serine. Even under serum‐free conditions, using only APE, ITS, and l‐ascorbic acid, the system demonstrated potential for large‐scale cell expansion. Yu et al. (2024) also reported that adding 12.5 µg/mL of *Grifola frondosa* extract ($0.51/g) to media containing 10% FBS significantly enhanced the proliferation and differentiation of bovine MuSCs, doubling the protein yield compared to 20% FBS. Nevertheless, challenges remain in standardizing and optimizing plant protein isolates. Batch variability can greatly impact cell culture outcomes; for instance, Stout, Rittenberg et al. (2023) reported up to a 3.5‐fold difference in cell numbers among different batches of rapeseed protein. Moreover, plant‐based alternatives may contain antinutritional factors, such as protease inhibitors and phenolic compounds, which can interfere with nutrient uptake or cell metabolism during extended cultures. Residual lectins in inadequately purified extracts can bind to cell membranes, disrupting signaling pathways or eliciting immune‐like responses (Samtiya et al. 2020). The long‐term functional equivalence of these alternatives is also yet to be fully established. To address these issues, it is crucial to establish consistent production processes, refine extraction methods to eliminate antinutrients, and conduct multi‐generational studies to validate long‐term performance.

#### 4.2.3 GF Stabilization and Immobilization

GFs are susceptible to degradation and inactivation under in vitro culture conditions. For instance, the half‐life of FGF2 in culture medium is only 6.3 h, leading to significant wastage (Ding and Peterson 2021). To address this, researchers have proposed immobilization techniques that anchor GFs to scaffolds, facilitating sustained and controlled release while preserving bioactivity essential for prolonged cultivation cycles. Bostock et al. (2022) successfully immobilized FGF2 in affibody hydrogels, significantly prolonging its release time. After 24 h, the FGF2 retained 30% of its activity and demonstrated improved thermal stability to prevent degradation. Additionally, scaffolds created by combining bioactive glass nanoparticles with polylactic acid sustained the release of FGF2 for 28 days. After 3 days of culture, these scaffolds showed a 20% increase in cell numbers compared to blank scaffolds (Yoon et al. 2017). Further research is required to determine whether immobilized GFs can maintain their biological activity over extended periods because cell numbers increased only 10% from Weeks 1 to 3 in the research. Further investigations could explore encapsulating additional macromolecular functional components within cell culture scaffolds to achieve effective and controlled release of these factors, representing a significant scientific opportunity.

#### 4.2.4 Circulation of SFM

The culture medium represents the most significant cost in CM production, accounting for 55%–95% of total expenses. This variability arises from differences in cell‐specific requirements, the concentration and type of GFs needed, the use of serum versus serum‐free formulations, and supplier pricing and production scales. For instance, Risner et al. (2020) demonstrated that producing CM using FGF2, priced at $2.05 million/g, results in a production cost of $400,000/kg of meat. Halving FGF costs could reduce expenses by up to 10‐fold. During cultivation, cells secrete autocrine/paracrine factors to sustain proliferation, yet a significant portion of proteins and nutrients remains underutilized. Therefore, effective removal of metabolic waste and recycling of residual nutrients are critical for cost‐effective, sustainable CM production, as they minimize resource consumption while enabling continuous high‐volume manufacturing processes. Cells primarily metabolize glucose and glutamine, but excessive byproducts such as lactate and ammonia threaten cell growth. Current strategies to mitigate these challenges include medium composition optimization, physical filtration, adsorption, and biocatalysis, though long‐term scalability remains constrained by technical limitations.

Medium composition optimization focuses on substituting or supplementing ingredients to reduce toxic byproduct accumulation. Yang et al. (2023) replaced glutamine with α‐ketoglutarate, which scavenges ammonia by forming glutamate. However, effective short‐term, prolonged α‐ketoglutarate use disrupts mitochondrial energy metabolism by altering flux through the tricarboxylic acid cycle, potentially impairing cell viability over successive passages. Similarly, copper ions (5 µM) have been shown to regulate gene expression, promote cell growth, and reduce lactate accumulation but exhibit cell‐type‐dependent efficacy (Qian et al. 2011). Chronic copper exposure induces oxidative DNA damage and epigenetic dysregulation, necessitating rigorous concentration control to avoid genotoxicity (Stepanyan et al. 2023). Such trade‐offs highlight the need for novel additives that balance metabolic efficiency with long‐term safety.

Physical filtration employs semipermeable membranes to separate waste molecules from reusable media components. Bernhardt et al. (2017) achieved a sevenfold improvement in cell viability over 28 days using cellulose dialysis membranes. However, membrane fouling and pore blockage during extended use necessitate frequent replacements, increasing operational costs. Adsorption‐based methods using materials like zeolite (60% within 24 h at 25 g/L) or α‐zirconium phosphate (four times more efficient than zeolite) offer higher ammonia removal efficiency but suffer from non‐specific ion competition, destabilizing critical signaling pathways (Kameda, Kikuchi et al. 2021; Yamaguchi et al. 2020). Recently, specific absorbents have been developed for targeted removal of lactate or ammonia. For example, Charalampopoulos's team used the weakly basic anion exchange resin Amberlite IRA67 to selectively remove 15% of lactate in neutral conditions (Zaini et al. 2019). Similarly, acidic cation exchange resins can remove ammonia from cell culture media, though inconsistencies in absorption conditions prevent efficient removal of waste. Combining multiple materials or using a stepwise approach has proven more effective for thorough waste removal. Kameda, Kikuchi et al. (2021) demonstrated that using 5 g/L Mg‐Al layered double oxides followed by 50 g/L strong acid cation exchange resin (PK216LH) removed 45% of lactate and 100% of ammonia.

Biocatalysis presents a promising alternative through enzymatic or microbial degradation of metabolic waste. Specific enzymes or microorganisms are introduced to break down or convert accumulated byproducts, maintaining a stable environment for cell growth. Specifically, lactate dehydrogenase oxidizes lactate to pyruvate, which is further metabolized to CO_2_ and H_2_O (Späte et al. 2024). Additionally, Haraguchi and Shimizu (2021) showed that treating C2C12 cell waste with *Centaurium littorale* reduced ammonia levels by 80%, while preserving essential inorganic salts like Ca^2+^ and Mg^2+^. However, residual microbial cells or endotoxins risk contaminating recycled media, potentially triggering immune activation or endotoxin shock in sensitive cell lines. Enzyme denaturation over repeated batches further reduces catalytic efficiency, necessitating costly replenishment. Innovative reactor designs may mitigate these issues: Shortall et al. (2023) immobilized thermostable aldehyde dehydrogenase and lactate dehydrogenase in a flow reactor, achieving 98% enzyme activity retention after 56 days of storage and repeated use without efficiency loss. Ultimately, scalable media recycling demands hybrid solutions that integrate rapid adsorption for initial waste removal with biocatalytic systems for sustained degradation. Addressing cumulative metabolic interference, material durability, and contamination risks will be pivotal to advancing CM toward industrial feasibility.

### 4.3 The Manufacture of Scaffolds

At present, the majority of CM production still relies on scaffolds for large‐scale production given the limited use of suspension cell lines in bioreactors (Ben‐Arye et al. 2020). Although scaffolds manufactured from natural sources such as animal, fungal, and plant materials have been extensively explored, non‐animal‐derived scaffolds that can efficiently support CM growth while remaining safe are still in their infancy of development. In this section, we provide an overview of recent advancements in CM scaffold design and material, aiming to offer valuable insights for the development of cost‐effective scaffolds that better meet the needs of CM production.

#### 4.3.1 Scaffolding Design Principles and Current Common Types

Scaffolds serve as templates for tissue formation, mimicking the extracellular matrix (ECM) to provide a 3D environment that supports cell growth, tissue formation, and spatial heterogeneity (Romani et al. 2021). They also provide biochemical and physical signals essential for CM development (Ben‐Arye and Levenberg 2019; Haraguchi and Shimizu 2021). CM scaffolds should balance functionality with affordability and scalability, adhering to the design principles of adhesion, mechanical strength, biocompatibility, and scalability, while also addressing specific requirements for degradability or edibility or removability (Table 3).

**TABLE 3:** Five core principles of scaffold construction.

Core principles	Description	Specific requirements
Adhesion	Considering the majority of the cell lines utilized for CM are anchored growth cells, scaffolds should allow cell adherence, ensure proper cell‐to‐cell contact, and support normal proliferation and differentiation (Bock et al. 2009)	Incorporate materials modified with RGD sequences or materials rich in RGD sequences, which bind to integrins in the cell (Derakhti et al. 2019; Ho et al. 2017)
Mechanical strength	Scaffolds must have sufficient stiffness to facilitate mechanical signal transduction and influence cell behavior (Frith et al. 2018)	Adjust scaffold stiffness to suit cell types: soft substrates (0.1–1 kPa) for osteoblasts, high stiffness (>34 kPa) for skeletal muscle cells (Humphrey et al. 2014)
Porous structure	Scaffold porosity determines cell interactions and the efficiency of nutrient and chemical signaling	Appropriate pore size can induce stem cells to differentiate specifically, for example, 50–150 nm for angiogenesis, >300 nm for osteogenesis, and 90–250 nm for cartilage formation (Ma and Huang 2020). Excessively low porosity can limit cell growth and differentiation (Humphrey et al. 2014), whereas excessively high porosity may reduce the mechanical strength of the scaffold and weaken its ability to interact with cells (Koushik et al. 2023)
Biocompatibility	Scaffold materials must not release harmful substances and should ensure cellular and tissue safety	Use non‐toxic, immunocompatible materials with long‐term cell implantation safety while minimizing cellular toxicity from degradation products
Palatability/Degradability/Removability	Scaffolds must be edible, biodegradable, or removable to avoid contaminating the final product (Bodiou et al. 2020)	Materials should naturally degrade, be easily removed, or integrate seamlessly into cultivated meat products without harmful residues

Abbreviation: RGD, arginine–glycine–aspartic acid.

**FIGURE 6:**
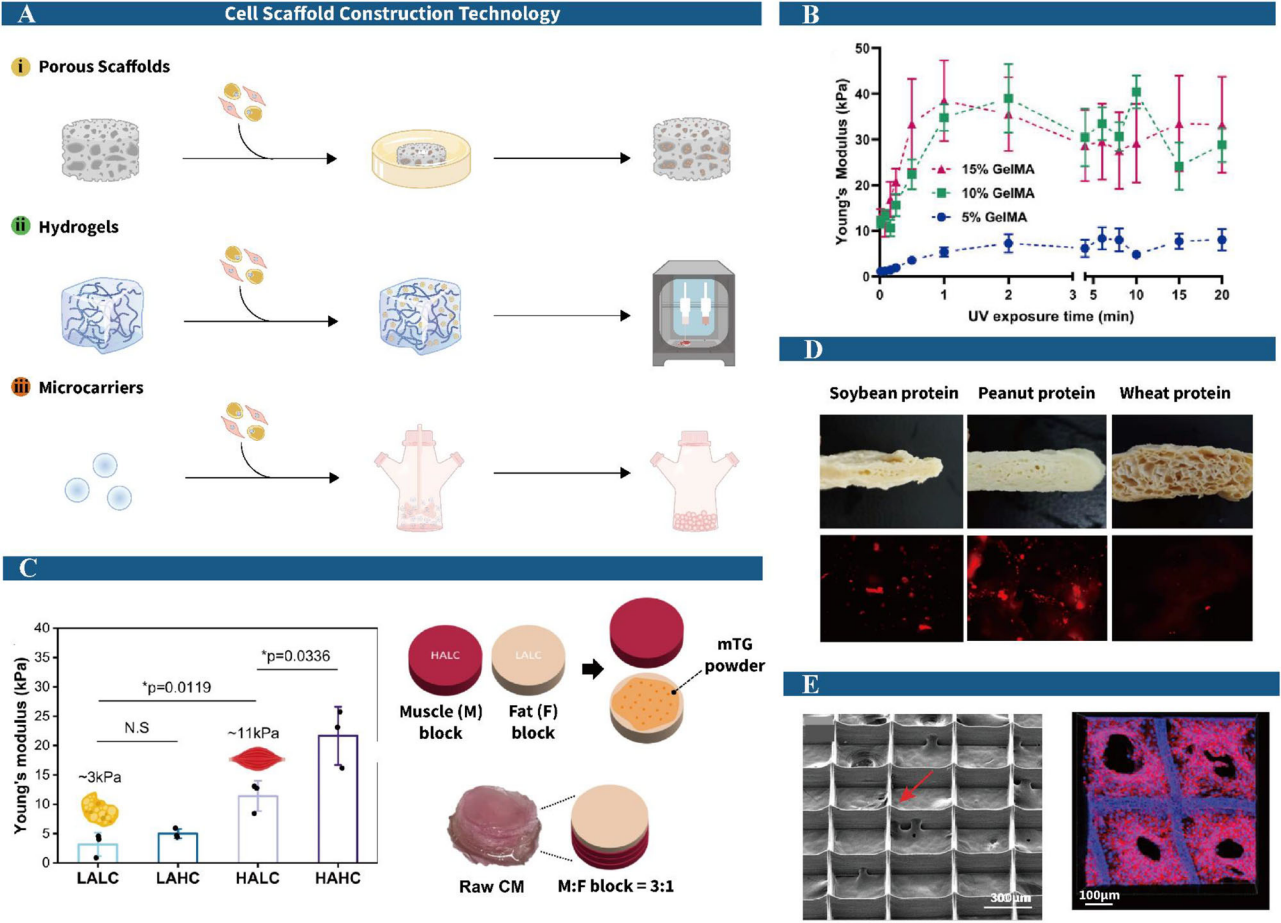
The cultured meat (CM) constructing techniques and materials for scaffolds. (A) Three types of scaffold forms for CM application techniques: (i) Porous scaffolds were placed in the culture medium after mixing the cells with the medium to allow the cells to adhere, proliferate, and differentiate to construct the structured CM; (ii) the cells and hydrogels were mixed to form bioinks, which were personalized to the shape of the meat using 3D printing, and then transferred to the petri dishes or perfusion bioreactors to proliferate and differentiate to construct the structured CM; (iii) after inoculating the cells to make the cells adhere to the microcarriers, the MCs were transferred to the stirred bioreactor, and the minced meat was finally collected by centrifugation; (B) young's modulus of gelatin can be regulated by the time of ultraviolet exposure (Smits et al. 2024); (C) hybrid hydrogels with adjustable stiffness were assembled to form beef blocks after inoculating cells separately (Lee, Park, et al. 2024); (D) the distribution of cells in the soybean, peanut, and gluten scaffolds was uneven, and the pore size of all three scaffolds was large (Zheng, Shi, et al. 2022); (E) combined with electrohydrodynamic printing technology, zein scaffolds are constructed with uniform pores, allowing cells to grow uniformly on them (Su et al. 2023).

Scaffolds for CM are commonly fabricated in three forms: hydrogels, PSs, and microcarriers (Figure 6A; Table 4) (Ben‐Arye and Levenberg 2019). Hydrogels, as crosslinked 3D polymer structures, closely resemble ECM in hydration and mechanical properties (Shi et al. 2019). Advanced techniques, including molding (Gu et al. 2024; Song, Liu, Li et al. 2022) and 3D printing (Chen, Dai et al. 2024; Xu et al. 2023), enable the creation of customized structured CM, such as marbled meats and layer‐by‐layer cultured fat and muscle (Li, Yang et al. 2022) or assembling them into marbled CMs (Zagury et al. 2022). More advanced studies have used fish muscle as a model using 3D printing combined with lipofilling to obtain customized cultured fish (Xu et al. 2023). However, these techniques, while demonstrating potential, are mainly applied to small‐scale production at high cost. PSs, known for their high porosity, expedite efficient nutrient exchange and waste removal (Hong and Do 2024; Zheng, Chen et al. 2022). Plant protein‐based PSs are cost‐effective but often lack the mechanical strength needed to support cell growth (Hollister 2005; Zheng, Chen et al. 2022). Techniques like freeze‐drying and electrospinning improve pore size and cell proliferation, with electrospinning emerging as a scalable method despite its material constraints, which include the need for polymers with appropriate viscosity, solubility, and electrostatic properties to ensure successful fiber formation (Xu et al. 2021; Kameda, Horikoshi et al. 2021). Decellularized plant scaffolds, which preserve the ECM and provide vascular‐like networks, are another innovative approach (Anjum et al. 2024). Examples include scaffolds derived from spinach leaves (Jones et al. 2021), celery (Hong and Do 2024), apples (Sood et al. 2024), pineapple nectar (Perreault et al. 2023), and maize husks (Perreault et al. 2023). Yet, prolonged use of these scaffolds can lead to nutrient depletion in the core, causing cell death (Xu et al. 2021). Although PSs have potential for large‐scale CM production, their mechanical strength and biological functionality, particularly in plant‐based materials, need further optimization (Zhang, Gao, et al. 2023).

**TABLE 4:** The manufacturing method of scaffolds.

Material type	Scaffold form	Scaffold fabrication	Material(s)	Crosslinking or treatment	Additive(s)	Highlights	References
Natural polymers							
Plant‐based	PSs	Freeze‐drying after molding	SA and cellulose	CaCl_2_	—	This scaffold supports bovine MuSCs proliferation and differentiation and mimics traditional meat's texture	Lee, Kim et al. (2024)
		Wet‐spinning	Car and SA	CaCl_2_	Single‐cell protein from mycelium	SA enhances CM cell adhesion and differentiation, especially with high CaCl_2_	Seo et al. (2023)
		Cutting	TVP			Myosin content and maturity are crucial indicators of quality, providing a valuable reference for quality control systems	Guan et al. (2023)
		Cutting	PWP	—	—	Low‐serum conditions boost ECM production in smooth muscle cells on PWP scaffolds, enhancing CM texture	Zheng, Chen et al. (2022)
		Cutting	PWP	—	—	Optimal cell density for adipogenesis and suitable PWP scaffolds yield cultured fat with improved volatile profiles but decreased texture properties	Song, Liu, Zheng et al. (2022)
		Freeze‐drying after molding	Wheat glutenin	—	—	Wheat glutenin scaffolds offer physical stability and support cellular activities, promising cost‐effective, food‐safe applications in CM	Xiang et al. (2022)
Natural polymers							
Plant‐based	PSs	Cutting	Soybean protein, wheat protein, and peanut protein	—	—	Long‐term subculturing reduces smooth muscle cell growth, whereas PWP supports adhesion and muscle protein secretion	Zheng, Shi et al. (2022)
		Decellularization	Celery	SDS	—	The study develops 3D chicken muscle‐like structures with contractile abilities using decellularized celery scaffolds, emphasizing scalable, cost‐effective protocols for sustainable CM production	Hong and Do (2024)
	Hydrogel	Mold	κ‐Car and KGM	KCl and CaCl_2_	—	This scaffold advances cultured fat production by optimizing cell growth and differentiation, matching natural fat's texture and nutrition	Gu et al. (2024)
		Mold	AlgMA and cRGD	Eosin yellowish, triethanolamine oxide, vinylpyrrolidone, EDC, NHS, CaCl_2_, and visible green light	—	Visible light and dual‐crosslinked SA hydrogels improve MuSCs’ viability, adhesion, and mechanical properties, advancing CM scaffolds	Tahir and Floreani (2022)
Natural polymers							
Plant‐based	Microcarriers	Electrospray microcarriers	SA, mung bean, soybean, red lentil, broad bean, pumpkin seed, rapeseed, chickpea protein, and pea protein	Trypsin treatment	—	Chickpea protein hydrolysates provide promising ECM‐mimic materials for CM, enhancing cell adhesion, proliferation, safety, and nutrition	Kong et al. (2023)
		Mold microcarriers	SA	CaCl_2_		This study demonstrated ADSCs’ adipogenic potential on MCs and proposed a method for expanding them for cultured fat production	Song, Liu, Li et al. (2022)
Animal‐based	Hydrogel	Mold	Collagen microfibers	Thrombin solution	Pristanic acid, phytanic acid, erucic acid, elaidic acid, oleic acid, palmitoleic acid, and myristoleic acid	This study explores optimizing CM's sensory properties using bovine ADSCs, aiming for scalable production focused on consumer acceptance and sustainability	Louis et al. (2023)
		DLP printing	GelMA	UV	—	Photocrosslinkable bioinks were optimized for 3D‐printed CM, enabling fibroblast differentiation into muscle and fat cells, supporting ethical, animal‐free production	Jeong et al. (2022)
		Mold	Rat tail collagen Type I	MTG and CaCl_2_		SMCs boosted collagen secretion and internal structure in CM, with hydrogel models improving texture after 6 days	Zheng et al. (2021)
Natural polymers							
Animal‐based	MCs	Water‐in‐oil emulsions grooved MCs	Gel	MTG	—	Edible MCs enable scalable, cost‐effective myogenic microtissue production, enhancing CM production and strengthening future food systems	Norris et al. (2022)
Hybrid	PSs	Freeze‐drying after molding	PC, DAC, collagen	—	Yeast proteins	Yeast protein replaces collagen in an edible scaffold, creating hypoallergenic, nutritious CM with beef‐like color and taste after frying	Wang, Zhong, Munawar, Zan et al. (2024)
		Freeze‐drying after molding	Tea polyphenols, SA, and Type A gel	—	—	SA‐gel scaffolds coated with tea polyphenols enhance cell adhesion, producing CM with rabbit MuSCs resembling real meat in appearance and texture	Chen et al. (2023a)
		Freeze‐drying after molding	PC, DAC, and collagen	—	—	PC and DAC enhance collagen stability and biocompatibility, preserving bovine myoblast stemness	Wang et al. (2023)
		Freeze‐drying after molding	PC, DAC, and collagen	—	—	These scaffolds improve porosity, biocompatibility, and mechanical properties, promoting myogenesis and producing beef‐like texture, color, and flavor	Wang, Zhong, Munawar, Wang et al. (2024)
		Freeze‐drying after molding	Gellan gum and Type A gel	CaCl_2_	—	This scaffold, enhanced by Ca^2+^ crosslinking, improves biocompatibility, cell proliferation, and myogenic differentiation, supporting scalable CM production	Chen et al. (2023b)
Natural polymers							
Hybrid	PSs	(a) Electrospinner nanofiber (b) Droplets of water‐in‐oil emulsions (c) Cutting	(a–b) Gel (c) Zein microfiber	(a) EDC‐HCl (b) MTG	—	Customized scaffolds for myogenic and adipogenic cells enable cohesive tissue constructs, mimicking marbled meat with ideal protein content	Kawecki et al. (2023)
		(a) Freeze‐drying after molding (b) Freeze‐drying after 3D printing	(a) SA‐RGD (b) SA‐RGD and pea protein	CaCl_2_ and sodium citrate	—	Cutured fat was engineered using MSCs in SA hydrogel, rapidly producing lipid‐rich tissue for integration with cultured muscle into cultured steak	Zagury et al. (2022)
	Hydrogel	Mold	Fish gel and SA	MTG and CaCl_2_	—	Scaffold engineering controls bovine MuSCs’ differentiation, producing CM with sensory and nutritional qualities comparable to traditional beef	Lee, Park et al. (2024)
		3D printing groove mold	Type A gel and SPI	MTG	—	3D‐printed scaffolds with 100–300 µm grooves optimize muscle gene expression and myotube development, enhancing cell functions on pea protein scaffolds	Chen, Han et al. (2024)
		3D printing groove mold	Soybean oil and gelMA	UV	LAP	Nano‐CM mimics real meat's structure and function, with aligned muscle and adjustable fat, offering advanced CM alternatives	Park et al. (2023)
Natural polymers							
Hybrid	PSs	Decellularization	Apple, Type A gel, and SA	CaCl_2_		The study presents an innovative CM method using biopolymer‐coated apple scaffolds to co‐culture bovine MuSCs and MSCs	Sood et al. (2024)
	Hydrogel	3D Printing	Gel, HA, silk fibroin, and chitosan	CaCl_2_	—	A 3D‐printed scaffold combined with fish gel for tissue‐like cultured fish fillets, enhancing cell differentiation and mimicking native muscle at scale	Xu et al. (2023)
		Coating and layer‐by‐layer assembly	(a) TVP, fish gel and agar (b) TVP, chitosan, CMC, fish gel, and agar	4°C freezing	(b) IGF‐1	A gel/agar coating for TVP was developed, creating CM that mimics traditional meat in size, texture, and flavor	Lee, Park et al. (2022)
		Layer‐by‐layer assembly	Chitosan, CMC, and sodium salt	EDC, NHS, and glutaraldehyde	C‐phycocyanin	This eco‐friendly platform promotes cell growth for CM, reducing serum use and potentially lowering production costs and complexities	Park et al. (2021)
	MCs	Electrospray MCs	Chitosan and collagen	TPP and EGCG	Chickpea protein dispersion, glycerol monostearate, and canola oil	A CM platform using edible MCs and oleogel‐based fat substitutes enables scalable production, mimicking traditional meat's texture, appearance, and nutrition	Yen et al. (2023)
**Natural polymers**							
		Electrospray MCs	Chitosan and bovine Achilles tendon collagen	TPP and EGCG	—	Edible cell MCs enhance CM production by simplifying harvesting, improving nutrition and texture, and promoting rapid cell proliferation	Zernov et al. (2022)
**Synthetic polymers**							
	Hydrogel	Microfluidic 3D printing	SA	CaCl_2_ and PVA	—	Coaxial microfluidic devices create core‐shell microfibers for CM, mimicking myofiber structure and promoting myogenesis	Ding et al. (2023)
**Scaffold free**							
		—	—	—	High‐moisture extruded pieces of soy protein, soy sauce, agave syrup, and tomato powder	Immortalized chicken fibroblasts showed stable phenotypes and high cell densities, contributing to potential cost reductions and scalability in CM production, with taste and sensory properties comparable to farmed chicken. Further work is needed to integrate different tissue types for enhanced texture	Pasitka et al. (2023)
		TRCD	Laminin‐511	—	—	The study successfully demonstrated the production of scaffold‐free CM using cell sheet technology, highlighting the need to enhance texture through methods such as collagen secretion and myotube formation	Tanaka et al. (2022)

Abbreviations: 3D, three dimension; ADSCs, adipose‐derived stem cells; AlgMA, methacrylated alginate; Car, carrageenan; CMC, carboxymethyl cellulose; cRGD, cysteine‐l‐arginyl–glycyl‐l‐aspartic acid; DAC, dialdehyde chitosan; DLP, digital light processing‐based; ECM, extracellular matrix; EDC, carbodiimide hydrochloric acid; EGCG, epigallocatechin gallate; gel, gelatin; gelMA, gelatin methacryloyl; HA, hyaluronic acid; IGF‐1, insulin‐like growth factor‐1; KGM, konjac glucomannan; LAP, lithiumphenyl‐2,4,6‐trimethylbenzoylphosphinate; MCs, microcarriers; MTG, glutamine transaminase; MuSCs, muscle stem cells; NHS, *N*‐hydroxysulfosuccinimide; PC, proanthocyanidins; PS, porous scaffold; PVA, polyvinyl alcohol; PWP, peanut wire‐drawing protein; RGD, arginine–glycine–aspartic acid; SA, sodium alginate; SDS, sodium dodecyl sulfate; SMCs, smooth muscle cells; SPI, soy protein isolate; TPP, tripolyphosphate; TRCD, temperature‐responsive culture dishes; TVP, textured vegetable protein; UV, ultraviolet.

Microcarriers, used in bioreactors, are ideal for large‐scale CM production, but they produce CMs that differ in appearance from traditional meat, making them better suited for processed meat products (Rodrigues et al. 2011). They are normally produced by techniques such as emulsification (Norris et al. 2022), electrospray (Yen et al. 2023; Zernov et al. 2022), and syringe extrusion (Andreassen et al. 2022). The efficiency of microcarriers depends on pore size and structure. Smaller pores (<10 µm) reduce cell attachment (Chen et al. 2011; Zernov et al. 2022), whereas larger pores (>10 µm) promote cell–cell interactions and improve nutrient transport (Ding et al. 2022; Huang et al. 2020). Studies have shown that pore diameters between 50 and 200 µm are most favorable for cell growth (Loh and Choong 2013). Besides, microcarriers with a diameter of <100 µm limit cell growth on curved surfaces, whereas larger diameters (500, 1500, and 3000 µm) increase the adhesion rate but decrease the proliferation rate (Bodiou et al. 2020). Designing the proper parameters is of vital importance in improving CM production efficiency.

Overall, although hydrogels and PSs show capability for structured CM production, reducing costs and enhancing their ability to support large‐scale tissue growth are ongoing challenges. Microcarriers, on the other hand, are better suited for unstructured CM but require further refinement in pore size and dimensions to increase cell proliferation. Future research should focus on improving these scaffold materials and techniques to boost production efficiency, lower costs, and support the commercial scalability of CM.

#### 4.3.2 Synthetic Polymer‐Based Scaffolds

Synthetic polymers offer the advantage of reproducibility, but their limitations in food safety and biocompatibility limit their suitability for CM applications (Gil et al. 2006). As a result, they are often used as auxiliary materials in scaffolds. Synthetic polymers primarily serve as structural supports, providing scaffolds with essential physical properties. For instance, Ding et al. (2023) developed a three‐layer microfluidic system where polyvinyl alcohol was incorporated into the inner layer to facilitate the crosslinking of SA with CaCl_2_. This integration also helped to regulate the viscosity and mechanical properties of the inner core, ensuring stability during microfiber formation.

Synthetic polymers can also function as temporary scaffolds that are later removed using temperature changes or electronic triggers (Moritz et al. 2015). For example, poly (*N*‐isopropylacrylamide), a temperature‐responsive material, is hydrophobic at temperatures above its low critical solution temperature (∼32°C, allowing cells to attach). When the temperature is lowered, the material becomes hydrophilic, causing the cells to detach (Tanaka et al. 2022). This property enables its use as a temporary carrier, with cell detachment achieved simply by reducing the temperature. Other methods, such as chemical or biological degradation, can also remove synthetic polymers. Specifically, ethylene diamine tetraacetic acid can dissolve Ca^2+^‐treated polygalacturonic acid, releasing the attached cells (Rodrigues, Rodrigues et al. 2019). However, these removal and degradation of synthetic polymers can impact cell viability and reduce yields.

From a large‐scale production standpoint, synthetic scaffolds can offer certain advantages. Their reproducibility and well‐established mass production techniques are beneficial for scaling up CM manufacturing. Additionally, synthetic scaffolds are cost‐effective, with materials such as polylactic acid and polycaprolactone priced under $10/kg, compared to natural materials like gel, which can cost up to $200/kg. But the need for additional steps to remove or degrade synthetic scaffolds, along with potential impacts on cell viability, diminishes these cost benefits. In this context, natural polymers that are biocompatible and edible emerge as a more desirable alternative. They eliminate the need for scaffold removal, simplifying the production process and potentially lowering costs associated with additional processing. Reducing the production costs of natural polymers or developing affordable, sustainable alternatives that retain the biocompatibility and functionality of natural materials may provide a viable path forward.

#### 4.3.3 Natural Polymers‐Based Scaffolds

##### 4.3.3.1 CM Scaffolds of Animal Origin

Animal‐derived polymers, such as collagen, gel, and silk fibroin, have garnered attention in CM research owing to their rich arginine–glycine–aspartic acid (RGD) sequences, which enhance cell adhesion (Gu et al. 2024; Jeong et al. 2022; Kawecki et al. 2023; Louis et al. 2023; Shams et al. 2022; Zou et al. 2022). Typically, pure animal protein scaffolds are formed into 3D network structures through crosslinking or chemical modification. Louis et al. (2023) used a combination of freeze‐drying, sonication, and thrombin to rapidly gel collagen. When bovine ADSCs were cultured with these scaffolds, the resulting fat had a composition, texture, and flavor similar to natural bovine fat. Nonetheless, collagen‐based networks tend to be softer, with limited crosslinking and low stiffness, making them appropriate for the growth of soft tissues such as fat or vascular structures (Jagiełło et al. 2022). In terms of large‐scale CM production, animal‐derived scaffolds pose several challenges. Their variability in quality and mechanical properties complicates standardization, and the processes required to modify these scaffolds to achieve suitable mechanical properties, such as crosslinking with methacrylic anhydride or using ultraviolet light (Figure 6B), raise concerns about residual chemicals and potential cytotoxicity (Smits et al. 2024). Although more recent approaches, such as enzymatic crosslinking using microbial transglutaminases, offer a safer and more robust alternative by avoiding harmful chemicals.

There is significant potential for optimization in reducing the cost of animal‐based scaffolds. One key approach is to improve the extraction and purification processes of animal protein like gel. Traditional extraction methods rely on acid, alkaline, or enzymatic treatments, but green technologies such as ultrasound‐assisted, microwave‐assisted, subcritical water extraction, and high‐pressure processing have demonstrated greater efficiency (Noor et al. 2021). These methods reduce energy consumption, shorten extraction time, and improve the properties of gel. As an example, ultrasound‐assisted extraction at 200 W and 60°C for 1 h achieves 1.2 times the efficiency of a traditional 5 h water bath at 37°C (Tu et al. 2015). Still, the reliance on animal materials presents ethical challenges and scalability issues owing to the need for consistent and sustainable sources of animal proteins (Norris et al. 2022). Advances in recombinant protein technology offer an alternative by introducing target genes into host cells, and optimizing culture conditions can enhance protein yield and purity (Wei et al. 2021). For example, yeast expression systems have been used to produce recombinant collagen with low endotoxin, high expression density, and cost‐effectiveness (Ramshaw et al. 2019). Although problems such as purification difficulties and low biological activity remain, advances in gene editing (e.g., the CRISPR technology), host engineering, and refinements in the efficient co‐expression of collagen and hydroxylase are expected to cut costs and elevate quality (Guo et al. 2024).

##### 4.3.3.2 CM Scaffolds of Fungal and Plant Origin

Fungal‐derived polysaccharides, particularly chitosan and its derivatives, are valued in CM production for their excellent biocompatibility, tunable mechanical properties, and biodegradability via lysozyme (Vallejo et al. 2022; Ren et al. 2018; Yang et al. 2020). Although chitosan can also be sourced from crustaceans (Dhanabalan et al. 2021; Rakshit et al. 2021) and insects (Kabalak et al. 2020; Poerio et al. 2020), fungal‐derived chitosan is favored for its efficiency and independence from seasonal fluctuations. Additionally, its positive charge enhances cell adhesion by interacting with negatively charged cells and serum substitutes (Park et al. 2021). Plant polysaccharides, including SA, hyaluronic acid, and carboxymethyl cellulose, are extensively applied in CM production in light of their low cost and availability (Tchobanian et al. 2019).

As these polysaccharides typically lack RGD sequences, their ability to support cell adhesion is restricted (Andersen et al. 2015). Chemical modification, material optimization, or modification by incorporation of animal proteins are often employed to increase cell affinity (Zernov et al. 2022). For instance, Tahir and Floreani (2022) modified SA by adding methacrylic anhydride and RGD peptides, creating a double‐crosslinked hydrogel using ultraviolet and Ca^2+^. This scaffold demonstrated significantly improved mitochondrial activity compared to standard gel scaffolds after 24 h of cell culture, indicating improved cellular performance. However, the high cost of RGD‐modified materials remains a barrier to widespread application. Another approach involves identifying natural materials that inherently support cell adhesion. Coarsely extracted SA, rich in glycoproteins, has been shown to facilitate cell attachment similarly to collagen scaffolds, with myoblasts maintaining a bipolar, elongated shape (Lee, Kim, et al. 2024). Specific polysaccharides, such as carboxymethyl cellulose and konjac glucomannan, facilitate cell adhesion due to their hydrophilic hydroxyl groups and lack of electrical charge (Hickey and Pelling 2019). Gu et al. (2024) developed a glucomannan‐based scaffold by heating at 95°C for 1 h, followed by cooling at 4°C overnight, forming a crosslinked network structure. However, effective for adipose tissue growth, this scaffold's low elasticity limits its use for muscle tissue. Studies suggest that rapid freezing (−28°C) could create smaller, more uniform pore structures, improving its potential for muscle tissue applications (Genevro et al. 2019). Hybrid scaffolds that combine plant‐derived materials with animal‐derived components offer another promising solution for overcoming these challenges, particularly in large‐scale CM production. Such scaffolds merge the biocompatibility and cell adhesion properties of animal‐based components with the mechanical strength and porosity of plant‐based materials, better mimicking natural tissues (Table 4). Lee, Park, et al. (2024) developed two tailored scaffolds: a low‐crosslinked SA/gel scaffold (Young's modulus: 3 kPa) for adipocytes and a high‐crosslinked SA/gel scaffold (Young's modulus: 11 kPa) for muscle cells. These scaffolds were later joined to form beef blocks and T‐bone steaks (Figure 6C). Moreover, hybrid scaffolds promote ECM secretion and render cells to modify the scaffold's microstructure through contraction, creating a complex cellular network that simulates the texture of the traditional meat (Zheng, Chen, et al. 2022). However, the high production costs and ethical concerns limit their use to niche applications where the unique properties of animal proteins are essential (Song, Liu, Li, et al. 2022; Tahir and Floreani 2022).

Instead, plant proteins such as peanut wire‐drawing protein, wheat gluten, and soy proteins offer advantages like low cost, edibility, and a porous texture that mimics traditional meat (Ben‐Arye et al. 2020; Zheng, Chen, et al. 2022). Processing conditions, including temperature, pressure, and humidity, induce interactions between protein molecules, forming stable scaffolds (Fonseca et al. 2021). For example, heating gluten promotes the formation of disulfide bonds and secondary structures like α‐helices and β‐sheets (Gil et al. 2006). It was demonstrated that 4% gluten could maintain the stable growth of bovine MuSCs for 14 days, and the fusion index was as high as 50% after cell induction and differentiation, symbolizing that gluten, a plant protein with a similar chewing sensation to traditional meat, can be used on CM (Xiang et al. 2022). Although most plant proteins lack cell adhesion sequences and have high porosity, certain proteins, such as those from pumpkin seeds, mung beans, soybeans, and chickpeas, contain RGD sequences that enhance cell adhesion (Kong and Huang 2023; Kong et al. 2023). Particularly, replacing gel‐coated microcarriers with proteins from broad beans, mung beans, or rapeseeds resulted in a four‐ to fivefold expansion of C2C12 cells within 3 days, whereas pumpkin seed proteins achieved a 6.8‐fold increase, comparable to gel (Kong and Huang 2023). Nonetheless, pure plant protein scaffolds still face challenges like uneven pore sizes, leading to inconsistent cell distribution (Figure 6D). Advanced techniques like electrospinning and 3D printing are utilized to create a uniform pore within the scaffold (Figure 6E) (Song, Liu, Zheng, et al. 2022; Zheng, Shi, et al. 2022), though they tend to increase production costs. Therefore, a scalable and cost‐effective approach is the combination of plant proteins with plant polysaccharides, which exploits the strengths of both materials to enhance scaffold performance while minimizing costs, making it a viable solution for CM production at scale.

#### 4.3.4 Other Scalable Scaffold Strategies for Cost‐Effective CM Production

Natural muscle fibers are highly organized, with cells aligned in specific directions to form muscle bundles. This alignment is guided by topographical cues in the ECM, which promote orderly cell growth. In contrast, smooth scaffold surfaces result in irregular cell adhesion, limiting growth and fusion rates (Huang et al. 2014). To mimic the fibrous structure of traditional meat while ensuring affordability, scalable micropatterning techniques and electrical stimulation systems can be integrated into manufacturing workflows, offering dual benefits of quality enhancement and cost efficiency.

##### 4.3.4.1 Topographical Cues

Achieving surface topography on biological scaffolds is essential for scaling up production, with both 2D patterned substrates and 3D scaffold microstructures offering viable solutions. 2D micropatterning methods, such as ion etching (Yamamoto et al. 2008) and soft lithography (Hosseini et al. 2012), enable precise alignment of muscle cells at minimal cost. For instance, microcarriers with 10 µm‐wide grooves increased C2C12 cell density by 1.6‐fold compared to spherical microcarriers, achieving scalable production without complex equipment (Norris et al. 2022). Although small curvature microcarriers may limit myotube fusion, optimizing groove dimensions reduces post‐culture time, thereby lowering labor and resource consumption (Werner et al. 2017; Li, Yang, et al. 2022).

3D microstructural technologies mimic native muscle environments more efficiently through multidimensional biomimetic designs that support cellular shape changes and ECM deposition. For instance, plant protein‐based scaffolds (e.g., pea or soy protein) fabricated via 3D printing with agar support baths allow low‐cost production of aligned fibrous scaffolds. This approach eliminates expenses for animal‐derived materials and enables mass production through automated printing (Ianovici et al. 2022). Additionally, directional freezing, a technique that controls cooling orientation during lyophilization, generates anisotropic pore structures, tripling scaffold compressive strength and enhancing cell differentiation efficiency (Jung et al. 2025). This process optimization minimizes material waste and shortens tissue maturation cycles, further reducing unit costs. Scalable methods, like electrospinning and wet spinning, utilize inexpensive polymers to produce high‐precision nanofiber scaffolds. Their rapid prototyping capabilities, high cell‐loading capacity, and elevated muscle marker expression reduce reagent consumption during cultivation (Kawecki et al. 2023; Su et al. 2023). The synergistic integration of these technologies marks a decisive leap toward industrial feasibility, collapsing per‐unit costs while preserving native tissue fidelity.

##### 4.3.4.2 Electrical Cues

Electrical stimulation offers a cost‐effective strategy to enhance CM production by accelerating cell maturation and reducing reliance on expensive GFs. By mimicking in vivo nerve impulses, electrical cues activate calcium signaling pathways, promoting muscle cell proliferation, migration, and differentiation (Stoppel et al. 2016). This process shortens tissue cultivation cycles, critical for scaling production, while improving muscle fiber alignment and mechanical properties. For large‐scale implementation, conductive materials, like polyaniline (Kumari et al. 2024; Zhang and Guo 2017), polypyrrole (Uzieliene et al. 2024), graphene (Adamowicz et al. 2020; Kim et al. 2020), and gold nanoparticles (Ge et al. 2021; Kim et al. 2019), can be integrated into scaffolds at minimal expense. Although non‐degradable materials like gold nanoparticles or graphene raise concerns about post‐harvest removal and expenses, degradable alternatives or conductive coatings provide scalable solutions. For example, polypyrrole‐coated scaffolds enhanced adipose‐derived stem cell differentiation into smooth muscle cells under low‐frequency stimulation (10 Hz), achieving functional maturation with minimal reliance on GFs or other costly additives (Björninen et al. 2017). These materials improve electrical conductivity while maintaining biocompatibility and low production costs. Further innovation, such as combining conductive coatings with temperature‐sensitive or enzyme‐responsive scaffolds, could enable automated scaffold removal post‐maturation, eliminating manual processing steps and further reducing operational costs. By optimizing electrical stimulation protocols and material choices, this approach balances performance enhancements with economic viability for industrial‐scale CM production.

### 4.4 The Application of Bioreactors

The transition from laboratory‐grade CM to industrial‐scale production demands a substantial supply of cells and a reliable, efficient production process. CM is typically produced in culture flasks, which have notable drawbacks, including low yield, slow production rates, and high costs. To facilitate affordable large‐scale production of CM, traditional cell culture methods must be optimized. Bioreactors, inspired by microbial fermentation technology, provide controlled conditions—such as temperature, pH, dissolved oxygen, and CO_2_—necessary for effective CM production (Petry and Salzig 2021; Specht and Lagally 2017). Research suggests that to lower CM production costs to below €10/kg, bioreactors with capacities of at least 100 m^3^ would be required. However, cells are typically cultured in bioreactors with volumes no greater than 20 m^3^ due to their high sensitivity to environmental variations (Kurt et al. 2022).

This bioreactor‐based process generally involves two stages: (1) seed cell culture, which focuses on maintaining the exponential growth of cells in nutrient‐rich environments while preventing differentiation, and (2) CM preparation, where seed cells are inoculated onto a scaffold to encourage their differentiation into mature CM. Therefore, efficient and cost‐effective production necessitates using multiple bioreactors in series alongside parallel production lines. Existing methods struggle to address issues such as nutrient and gas gradients in large‐scale environments and excessive shear forces that can damage cells. Although cell suspension culture techniques have been used in CM production, there are ongoing challenges with structured CM.

#### 4.4.1 Cell Suspension Culture Approaches

Dynamic bioreactors are frequently employed to enhance cell yields by cultivating cells in suspension. This process involves either inoculating cells onto suspension‐capable carriers or converting adherent cells into suspension cultures. The most common approach is to utilize microcarriers into bioreactors, which is relatively straightforward (Andreassen et al. 2022; Bodiou et al. 2020; Zernov et al. 2022). Microcarriers facilitate the efficient transport of oxygen, nutrients, and metabolic waste, thereby promoting cell growth (Huang et al. 2020; Meng et al. 2017). Verbruggen et al. (2018) demonstrated that culturing bovine myoblasts on microcarriers in spinner flasks enhances both growth and mass production. Nevertheless, it is notable that microcarriers occupy a significant portion of the bioreactor space. If they are inedible, they may limit the amount of CM harvested. Even edible microcarriers can affect dissolved oxygen levels, nutrient distribution, and medium viscosity, which ultimately impacts the quality and efficiency of large‐scale CM production (Sousa et al. 2015). Addressing how microcarrier movement and distribution influence the bioreactor environment in large‐scale, high‐density cultures is essential for optimizing production.

Cell adaptation techniques offer a sophisticated method for transforming adherent cells into suspension‐adapted lines, which is crucial for large‐scale CM production. This approach not only advances bioreactor scalability but also removes the constraints associated with microcarriers, reducing production costs. It enhances flexibility and automation, making the production process more responsive to market demand. The cell suspension adaptation process involves four key steps. First, cells are immortalized and selected or induced to adapt to suspension culture (Zhang, Qiu, et al. 2023). Second, non‐adherent cell spheres are formed to simulate the suspension environment, decreasing reliance on attachment (Busch et al. 2023). Third, after prolonged stirring, cell aggregates are gradually broken down into single cells, with only those exhibiting mechanical resilience surviving shear stress and continuing to proliferate (Pasitka et al. 2023). Finally, stable cell clones capable of thriving in suspension are selected through repeated rounds of multiplication. For example, Pasitka et al. (2023) successfully transformed chicken fibroblasts for suspension culture, achieving 1.5 years of continuous culture and over 600 cell divisions. This method harvested the large‐scale production of cultured fat at 360 g/L, representing a significant improvement over other reported CM techniques. Nonetheless, the reliance on genetic engineering techniques necessitates a thorough examination of their safety. Additionally, pure cell‐derived CM may have a different taste compared to conventional meat due to the absence of ECM.

#### 4.4.2 Engineering of Bioreactors

A stable and efficient culture medium not only provides essential nutrients but also combines mechanical engineering with cell biology to maintain pH stability and ensure an adequate oxygen supply, all essential for high‐density CM production. Bioreactors serve as intelligent devices that simulate the in vivo environment, adapting to the cellular needs. Insufficient CO_2_ during early cell proliferation or excessive CO_2_ during exponential growth can disrupt the partial pressure of CO_2_ in the medium, impeding aerobic metabolism. In high‐density cultures, CO_2_ is produced when the respiratory quotient is 1, whereas lactic acid is generated when it exceeds 1. Both byproducts affect the medium's pH and hinder cell growth. Optimizing oxygen, CO_2_, and buffering systems in large‐scale production is essential for maintaining cellular metabolism. Mammalian cells generally require dissolved oxygen levels between 30% and 50%, but supplying sufficient oxygen in large‐scale bioreactors is challenging due to oxygen's low solubility in water (Zhang et al. 2015; Sieblist et al. 2016). Research shows that agitation improves gas dispersion, with faster agitation enhancing gas transfer and the volumetric mass dissolved oxygen transfer coefficient (Umar et al. 2023). Agitation‐based bioreactors (ABs) are well‐suited for supplying oxygen to CM cells in high‐density cultures, but the trade‐off between high agitation speeds and significant shear stress on cells requires careful optimization.

Bioreactors can be classified into agitation‐based and perfusion‐based types. Studies show that ABs can yield over five times more CM cells than PBs under similar conditions (Cameron et al. 2006; Specht and Lagally 2017). These include stirred, wave, and airlift types, which generally achieve higher CM yields, although they are less conducive to forming structured CM. Fluid movement in these systems can inversely lead to mechanical damage to cells, thereby reducing yields (Berry et al. 2016). To mitigate this damage, agitation experiments typically restricted stirring rates to 40–120 RPM. This limitation, however, can decrease gas–liquid mass transfer efficiency and liquid‐phase mixing, ultimately reducing CM yield and quality (Meng et al. 2017). Higher agitation speeds, specifically 300 or 325 RPM, have demonstrated an enhancement in CM yields, indicating that shear stress may be less detrimental than deficiencies in nutrients or oxygen, contingent upon the cell type (Melke et al. 2018; Pasitka et al. 2023). Intermittent stirring has also been suggested as a method to mitigate cell damage. Following 3 h of continuous agitation and 45 min of intermittent stirring, cell volumes nearing those of skeletal muscle (with a density of 10^8^/cm^3^) were achieved, resulting in a 2.9 g CM harvest from a 100 mL rotating flask (Norris et al. 2022). The airlift bioreactor demonstrates superior medium homogeneity, minimal power requirements, and reduced shear stress, positioning it as a viable candidate for large‐scale cell culture (Li et al. 2020). Yet, its application remains constrained to computer simulations, necessitating additional empirical research to evaluate its feasibility for CM production. Wave bioreactors offer advantages over stirred and airlift bioreactors, including ease of operation, low shear stress, reduced risk of cross‐contamination, minimal cellular damage, and improved oxygen and nutrient transfer. Still, they are usually miniaturized, and their potential for large‐scale application remains to be fully explored (Godfray et al. 2018). Recently, Mercedes and Teresa (2023) introduced a scalable wave bioreactor system integrating movable grids and staged culture volumes. Their design first seeds 3D scaffolds in a 5%–20% volume seeding chamber (5000–25,000 cells/cm^2^), followed by incubation in expanded volumes via grid repositioning. This approach maintained pH 7.0–7.4 and 30%–60% dissolved oxygen over 10–60 days, demonstrating adaptability for structured CM production while addressing scale‐up challenges of nutrient gradients and shear stress.

PBs eliminate foam generation on account of their stable energy input, thereby reducing shear stress on cells (Werner et al. 2010). They are categorized into three main types: hollow‐fiber bioreactors, packed‐bed bioreactors, and fixed‐bed bioreactors. Hollow‐fiber bioreactors mimic the vascular network, efficiently supplying nutrients and removing metabolic waste, yet struggle with fluid distribution, mass transfer, gas exchange, and waste disposal in large volumes, increasing costs (Guan et al. 2022). Packed‐bed and fixed‐bed bioreactors support cell adhesion but may develop “dead zones” with uneven fluid distribution, resulting in localized cell growth limitations or cell death (Mostafavi et al. 2021). Morel et al. (2024) designed a dynamically adjustable fiber scaffold integrated within the bioreactor, which actively improves nutrient distribution and metabolic waste removal by real‐time flow‐path optimization, thereby minimizing dead zones. Currently, PBs are confined to millimeter‐scale scaffolds, with scaling to industrial volumes remaining unfeasible (Chen et al. 2022; Jaasma et al. 2008). For example, producing 1 kg of CM may require a 40 L bioreactor (Norris et al. 2022), and to meet global demand for meat, approximately 23 billion L of bioreactor capacity would be required daily (0.5 kg of meat per person per week). Existing industrial bioreactors have volumes of only 2.5 L, making it impractical to scale up through multiple smaller reactors (Datar and Betti 2010).

The selection of bioreactors for CM production requires balancing yield, product quality, and cost‐effectiveness, guided by specific operational goals. Yield‐driven unstructured CM (e.g., minced meat) favors ABs for their high throughput and cost efficiency, despite shear‐related compromises. Stirred bioreactor dominates here, leveraging scalability and adaptable agitation protocols. For structured CM requiring 3D tissue architecture, PBs are indispensable despite higher operational costs and technical complexity, as they enable nutrient gradients and cell alignment akin to native muscle. Intermediate solutions like airlift bioreactors balance shear reduction and energy efficiency for moderate‐scale operations, whereas wave bioreactors suit pilot‐scale research and development. To address global demand, future innovations must prioritize scalable PB systems (e.g., modular perfusion units) and hybrid systems integrating AB efficiency with PB structural fidelity. Advancements in adaptive fluid dynamics (e.g., artificial intelligence‐driven shear stress modulation) and biomimetic scaffold designs will be pivotal in bridging the gap between industrial feasibility and product quality.

## 5 Integrated Technologies for Scalable and Affordable CM Production

The industrial‐scale production of CM necessitates coordinated innovations from multiple disciplines, including cell biology, bioprocess engineering, and materials science. Cost reduction and achieving large‐scale production are highly interconnected: The former is achieved through technological innovations that lower unit costs, whereas the latter is realized through process amplification that ensures economic feasibility. At the same time, pursuing cost control while ensuring that large‐scale expansion maintains both biological activity and process stability presents a significant dual challenge. This section delineates the critical role of integrated technological frameworks in driving economic feasibility and operational reliability, followed by strategies to harmonize cost efficiency with stability during scale‐up.

### 5.1 The Strategic Imperative of Technology Integration

Analysis of the four technologies above indicates that achieving cost parity with conventional meat requires the integration of three core technological advancements. To begin with, eliminating animal components from cell cultures offers the greatest cost‐saving opportunities. For instance, recombinant protein or plant‐based substitutes can slash GF costs by over three orders of magnitude, therefore removing them as cost drivers for CM and decreasing CM production costs from $22,421/kg to $116/kg. Additional cost reductions to $18/kg can be attained through a 100‐fold decrease in expenditures on recombinant albumin (Vergeer 2021). Second, in cell engineering, self‐sustaining cell systems designed through autocrine/paracrine signaling amplification or endogenous GF secretion have markedly reduced reliance on exogenous proteins, thereby lowering costs and simplifying large‐scale production processes. Research also indicates that improved proliferation kinetics, exemplified by a 25% reduction in running time, can result in an 11% cost reduction. Additionally, increasing cell volume to 5000 µm^3^ can yield a further 10% savings through optimized biomass yield, enhancing the efficiency of large‐scale bioreactors (Vergeer 2021). Third, optimizing the design of strategic bioreactors is essential for managing capital expenditures. The investment for 50 L stirred bioreactors is $20 million; however, scaling to 10,000 L stirred bioreactors and 2000 L PBs increases costs to $150 million and $260 million, respectively, necessitating more advanced engineering solutions (Vergeer 2021). Future Meat Technologies Co. has demonstrated that tangential flow filtration bioreactors can achieve 43% higher volumetric productivity than traditional systems, leading to a production cost of about $6.20/pound when scaled to 5000 L operations (Pasitka et al. 2023).

Leading commercial platforms illustrate these advancements. The estimated cost of $6.20/pound of cultured chicken is derived from the use of suspension‐adapted cell lines (1.3 × 10^11^ cells/L), low‐cost SFM at $0.63/L, and high‐efficiency bioreactors (Pasitka et al. 2023). In parallel, the Kaplan team's CRISPR‐engineered bovine myocytes, which secrete endogenous FGF2, show promising potential for decreasing GF costs by 90% (Stout, Zhang et al. 2023). Their innovation also includes cost‐effective SFM utilizing non‐hydrolyzed rapeseed proteins as recombinant protein alternatives (Stout, Rittenberg et al. 2023). Negulescu et al. (2023) performed a techno‐economic analysis based on large‐scale bioreactors. To achieve a cultured beef cost below $9/kg, a cell density of at least 3.3 × 10^7^ cells/mL is required. Additionally, it should integrate a 211,000 L stirred bioreactor with reduced media costs of $0.45/L, or a 262,000 L airlift bioreactor with lower media costs of $0.75/L. Humbird (2021) indicated that substantial increases in production scale could lead to nutrient costs nearing zero. The utilization of hydrolysates at a cost of $2/kg, combined with fed‐batch processes, allows for a reduction in macronutrient expenses by $16/kg, resulting in a total production cost of $22/kg.

In summary, integrated technologies for scalable and affordable CM production include three key areas: (1) substitution of animal‐derived components with plant‐based or recombinant alternatives, (2) development of self‐sustaining cellular systems through genetic and metabolic engineering, and (3) deployment of advanced bioreactor strategies. Their deep interconnectivity creates a self‐reinforcing innovation cycle, making CM commercialization economically viable. Progress in cell line engineering can minimize reliance on GFs or enable the development of scaffold‐free suspension cell lines. Plant‐based substitutes and recombinant proteins further cut the cost through SFM and advanced scaffold materials. Cost‐effective plant alternatives such as rapeseed and pumpkin protein facilitate cell growth and adhesion, serving as substitutes for animal‐derived components. Recombinant systems, including microbial and plant‐based, generate recombinant proteins that exhibit comparable mechanical properties and biocompatibility. Research into cellular metabolism optimizes nutrient utilization, aligning with bioreactor programming to achieve low‐energy, cost‐efficient cultivation. Media recycling and reuse contribute to both economic and environmental sustainability, whereas understanding scaffold behavior in bioreactors aids in developing customized approaches for tissue growth.

### 5.2 Stability Assurance in Cost‐Optimized Scaling

Engineering industrial‐grade CM bioprocesses demands systematic stability frameworks to navigate both biological and technological components. Significant challenges involve maintaining cellular functionality, ensuring the consistency of plant‐based or recombinant inputs, and guaranteeing aseptic production. Here, we delineate potential approaches for achieving stability during scale‐up.

For cellular system stability, maintaining the stability of cellular systems is crucial for scalable CM production. Alongside the selection of cell lines exhibiting stable genotype and phenotype characteristics, the optimization of cell culture conditions and the implementation of long‐term monitoring are vital. Computational tools such as mathematical modeling and response surface methodology can analyze and quantify the complex interactions between technical variables, discovering optimal parameter combinations (Rodrigues, Fontão, et al. 2019). Plus, machine learning and deep learning technologies can optimize non‐linear and multidimensional variables, allowing for precise adjustments to factors such as culture medium formulations and cell growth conditions (Safford et al. 2024; Konishi 2024). The integration of these technologies with dynamic modeling and process simulation, including kinetic models, facilitates the analysis and optimization of the entire production process (Xiao et al. 2024). Recent innovations by Ranger et al. (2024) integrate high‐frequency ultrasound probes (up to 100 MHz) with machine learning algorithms to monitor cellular confluence and differentiation in real‐time. This system enables closed‐loop control of wave‐induced fluid dynamics, maintaining >90% cell viability while accommodating perfusion volumes of up to 2000 L, representing a significant advancement in CM production. Moreover, routine assessments of genomic stability through techniques like chromosomal analysis and genome sequencing are essential. The introduction of resistance genes or selective marker genes into cell lines can also enhance the maintenance of genetically stable cell populations and decrease the prevalence of unstable cells (Ng et al. 2017).

For the stability of plant‐based or recombinant components, ensuring batch‐to‐batch consistency of plant‐derived and recombinant substitutes requires stringent quality control measures for the stability of these components. Multi‐stage chromatographic purification and mass spectrometry fingerprinting help minimize variability from raw material sources, such as soybean or rapeseed cultivars (Donno et al. 2020). Machine learning platforms can accelerate the functional validation of plant protein analogs by predicting their receptor‐binding equivalence to animal‐derived counterparts, reducing the necessity for experimental iterations (Panahi et al. 2024).

Aseptic production requires sterility in raw materials and the production environment. Although CM production requires food‐grade materials, the need for sterile conditions, especially due to the involvement of animal cells, elevated the associated instability and cost. High‐temperature sterilization is frequently employed; however, proteins in culture media or scaffolds may experience heat denaturation. Enhancing thermal stability, such as by designing heat‐stable protein variants with engineered disulfide bonds, can improve resilience during sterilization. For instance, α‐amylase that has been modified to include engineered disulfide bonds exhibits a 40‐fold increase in half‐life, approximately 23 h, at 90°C (Zhu, Zhai, et al. 2023). Furthermore, existing production facilities for CM are required to adhere to ISO 8 cleanroom standards, which may restrict the scalability of bioreactor operations and consistency between batches (Goodwin et al. 2024). It is essential to establish formal standards and regulations for sterile production in food‐grade contract manufacturing facilities.

Combining these advanced technologies with standardized production systems, automation, and modularity lays a solid foundation for the industrialization of CM. Driven by the seamless integration and continuous innovation of core technologies, CM production is expected to establish a scalable, economically feasible, and sustainable system that not only meets the growing global demand but also becomes an essential component of the future food supply chain.

## 6 Conclusions

The future of cost‐effective, large‐scale CM production depends on the seamless integration of cell lines, culture media, scaffolds, and bioreactors into a unified system. These core technologies are deeply interconnected, with advances in one area directly enhancing the others. This synergy is crucial for scaling up from laboratory production to efficient, industrial‐scale systems. Key advancements in these technologies center around three primary areas. First, cell line engineering is foundational to enabling scalable production. The development of genetically stable, highly expandable immortalized cell lines through advanced gene‐editing techniques, such as CRISPR, reduces the need for frequent seed cell extraction and provides a strong basis for mass production. Additionally, engineering cells to enhance autocrine and paracrine signaling reduces reliance on expensive exogenous GFs, addressing a major cost bottleneck. Adapting cells for suspension culture further simplifies bioreactor operations, reduces the need for scaffolds, and enhances scalability and flexibility in production. Second, innovations in plant‐based substitutes and recombinant protein technologies are vital for both SFM development and scaffold fabrication. Cost‐effective alternatives like rapeseed‐derived plants replace animal‐based components and support efficient cell growth. Similarly, certain plant proteins, such as pumpkin protein, exhibit promising cell adhesion properties, enabling their use in scaffolds as substitutes for animal‐based proteins as well. In parallel, host systems, such as plant or microbial, enable the production of recombinant proteins and scaffold materials with enhanced mechanical properties and biocompatibility, such as recombinant collagen combined with plant polysaccharides. Finally, bioreactors, the core infrastructure for CM mass production, require innovation to balance efficiency with cost‐effectiveness. Closed‐loop systems that recycle and reuse media, tailored to the metabolic demands of cells, enhance nutrient utilization while reducing energy consumption and waste. Dynamic environmental control within bioreactors, including optimized oxygen and CO_2_ delivery and low‐shear stress conditions, ensures cell viability and supports high‐density cell cultures. Additionally, understanding the behavior of scaffolds in dynamic environments enables better coordination between scaffold materials and bioreactor operations. Scaling bioreactors for industrial production demands overcoming challenges related to oxygen and nutrient gradients, shear stress, and operational complexity, necessitating the design of specialized systems like stirred or airlift reactors to maximize productivity. With the support of strategies such as dynamic modeling, computational simulations, and automation technologies, these four core technologies can be further optimized and deeply integrated. By establishing modular, standardized production workflows, the CM industry is poised to create an efficient, cost‐effective, and sustainable system. These advancements will boost its competitiveness in the global market and could revolutionize sustainable food production, offering a viable alternative to traditional meat systems.

## Author Contributions

**Huiwen Gu**: writing – original draft, visualization, conceptualization, software, methodology, data curation, investigation, validation, formal analysis and writing – review and editing. **Yan Kong**: data curation, supervision, writing – review and editing and funding acquisition. **Dejian Huang**: formal analysis, writing – review and editing and resources. **Youfa Wang**: conceptualization, writing – review and editing and supervision. **Vijaya Raghavan**: writing – review and editing. **Jin Wang**: conceptualization, methodology, formal analysis, supervision, funding acquisition, project administration, resources, writing – review and editing and visualization.

## Conflicts of Interest

The authors declare no conflicts of interest.

## Supporting information




**Supplementary Tables**: crf370221‐sup‐0001‐SuppMat.docx
