# Supplementary material for: Scaling Cultured Meat: Challenges and Solutions for Affordable Mass Production
Source: Compr Rev Food Sci Food Saf. 2025 Jul 9;24(4):e70221. doi: 10.1111/1541-4337.70221 (PMC12241508; doi:10.1111/1541-4337.70221)
Supplement: Supplementary file 1 — Supplementary Tables: crf370221‐sup‐0001‐SuppMat.docx [file CRF3-24-e70221-s001.docx]

**Scaling Cultured Meat: Challenges and Solutions for Affordable Mass Production**

Huiwen Gu^a^, Yan Kong^a,b^, Dejian Huang^c^, YouFa Wang^a^, Vijaya Raghavan^d^, Jin Wang^a,d^*

^a^ Key Laboratory of Environmental Medicine and Engineering, Ministry of Education, and Department of Nutrition and Food Hygiene, School of Public Health, Southeast University, Nanjing 210009, China

^b^ National University of Singapore (Suzhou) Research Institute, 377 Lin Quan Street, Suzhou Industrial Park, Jiangsu, Suzhou, 215123, China

^c^ Department of Food Science and Technology, 2 Science Drive 2, National University of Singapore, 117542, Singapore

^d^ Department of Bioresource Engineering, Faculty of Agricultural and Environmental Sciences, McGill University, 21111 Lakeshore Rd, Sainte-Anne-de-Bellevue, H9X3V9, QC, Canada

***Correspondence author**

Prof. Dr. Jin Wang, [jinwang_2020@seu.edu.cn](mailto:jinwang_2020@seu.edu.cn)

<https://orcid.org/0000-0003-3117-9173>

Department of Nutrition and Food Hygiene, School of Public Health, Southeast University, Nanjing 210009, China

Department of Bioresource Engineering, Faculty of Agricultural and Environmental Sciences, McGill University, 21111 Lakeshore Rd, Sainte-Anne-de-Bellevue, H9X3V9, QC, Canada

**Short version of title (running head)**

Cultured Meat Scaling: Challenges and Solutions

**SUPPLEMENTARY 1** The flavor analysis of various cultured meat (CM)

| **Type** | **Method** | **Flavor Results** | **Reference** |
| --- | --- | --- | --- |
| Cultured beef | GC-MS | CM and beef share Maillard reaction compounds and similar flavors like almond and fat, with CM's high protein and lipid contents mimicking beef's flavor profile. | (Lee et al., 2024b) |
|  | Electronic tongue | Minor taste differences exist between CM and beef, with YP10 and YP30 CMs tasting more similar to beef than the collagen variant. | (Wang et al., 2024c) |
|  | Electronic tongue | CM has a similar flavor profile to beef, with slight differences in specific tastes, suggesting a comparable sensory experience. | (Wang et al., 2024b) |
|  | Electronic nose | The fishy odor-causing compounds are effectively removed during the CM manufacturing process. | (Lee et al., 2024a) |
| Cultured pork | GC-MS | Cultured pork's volatile profile resembles porcine tissue but varies in compounds like hexanal and 2-heptanone from peanut scaffolds, affecting pork flavor. High benzaldehyde in cultured pork, impacting aroma, underscores the need for flavor optimization. | (Song et al., 2022a) |
| Cultured mouse | GC-MS | Upon grilling, CM experienced a Maillard reaction, with its Gel and agar coatings and myoblasts enriching the flavor with savory, oily, and meat-like notes to more closely resemble that of traditional meat. | (Lee et al., 2022a) |
| Cultured chicken | Volunteer taste test | Cultured chicken, augmented with adipocyte-like cells, received higher flavor ratings than its soy base, was preferred by most, and showed strong potential as a viable meat substitute. | (Pasitka et al., 2023) |

**Abbreviations:** GC-MS, Gas Chromatography–Mass Spectrometer; YP, Yeast Protein; Gel, gelatin

**SUPPLEMENTARY 2** The nutrient composition of various cultured meat (CM)

| **Type** | **Scaffold** | **Moisture (g/100g)** | **Carbohydrates (g/100g)** | **Fat (g/100g)** | **Protein (g/100g)** | **Reference** |
| --- | --- | --- | --- | --- | --- | --- |
| Cultured beef | Hybrid scaffold |  | Raw CM: 3  Raw beef: 7.97 | Raw CM: 0.137  Raw beef: 6.7 | Raw CM: 10.71  Raw beef: 19.88 | (Lee et al., 2024b) |
|  |  | Raw CM: ≈90  Raw beef: ≈78 |  | Raw CM: 0.2-1.2  Raw beef: ≈1.85g | Raw CM: 12-13  Raw beef: ≈14 | (Wang et al., 2024c) |
|  |  |  | Blank scaffold: ≈1.01 |  |  | (Lee et al., 2024a) |
|  |  | Raw CM: 93.30-95.49  Raw beef: ≈72.19 |  | Raw CM: 0.135-0.600  Raw beef: ≈1.27 | Raw CM: 11.13 -14.00  Raw beef: ≈14.24 | (Wang et al., 2024b) |
|  |  | Raw layered CM: ≈75  Raw burger-like CM: ≈50  Raw beef: ≈75 |  |  |  | (Yen et al., 2023) |
|  | Scaffold free | Raw CM (D1): ≈87.5%  Raw CM (D7): ≈88.1%  Raw beef: ≈60.0% | Raw CM (D7):  ≈1.3% (wet weight)/  ≈10.7% (dry weight)  Raw beef:  ≈0.9% (wet weight)/  ≈2.2% (dry weight) |  | Raw CM (D7): ≈42.3%  Raw beef: ≈28.8% | (Tanaka et al., 2022) |
| Cultured pork | Plant-based scaffold | Raw CM: ≈80.24  Raw pork: ≈72.19. |  | Raw CM: ≈0.6  Raw pork: ≈2.2. | Raw CM: ≈15.22  Raw pork: ≈20 | (Guan et al., 2023) |
|  |  | Blank scaffold: ≈81.12  Raw CM: ≈81.35  Raw pork: ≈86.08 |  | Blank scaffold: ≈0.38  Raw CM: ≈0.15  Raw pork: ≈1.92 | Blank scaffold: ≈9.95  Raw CM: ≈10.9  Raw pork: ≈12.79 | (Zheng et al., 2022a) |
|  | Animal-based scaffold |  |  |  | Raw CM Collagen (D9): 0.27 | (Zheng et al., 2021) |

**Refer to SUPPLEMENTAY 2 (continued)**

| **Type** | **Scaffold** | **Moisture (g/100g)** | **Carbohydrates (g/100g)** | **Fat (g/100g)** | **Protein (g/100g)** | **Reference** |
| --- | --- | --- | --- | --- | --- | --- |
| Cultured rabbit |  |  |  |  | Raw CM: 4  Raw Wagyu :9 | (Kawecki et al., 2023) |
| Cultured Fish | Hybrid scaffold |  |  | Blank scaffold: 0  Raw CM: 0.9  Raw fish: 1.3 | Blank scaffold: 9.6  Raw CM: 18.4  Raw fish: 19.9 | (Niu et al., 2025) |

**Abbreviation:** CM, Cultured Mea**t**

**SUPPLEMENTARY 3** The seed cells and the corresponding medium applied in cultured meat (CM)

| **Class** | **Genus** | **Seed cells** | **Growth Medium** | **Differentiation Medium** | **Reference** |
| --- | --- | --- | --- | --- | --- |
| Mammalia | Bovine | Myoblasts | LG-DMEM supplemented with 10% FBS and 1% AB/AM | HG-DMEM supplemented with 5% HS and 1% P/S/G | (Lee et al., 2024b) |
|  |  |  | DMEM/F12 containing 20 % FBS and 1 % P/S | DMEM/F12 supplemented with 2 % HS and 1 % P/S. | (Wang et al., 2024c) |
|  |  | MuSCs | DMEM containing 10 % FBS and 100 units/ml P/S | DMEM supplemented with 1% FBS, 1 μM insulin, and 100 units/ml P/S | (Lee et al., 2024a) |
|  |  |  | DMEM containing 20 % FBS and 1% P/S/G | - | (Sood et al., 2024) |
|  |  |  | DMEM containing 10 % FBS and 1% AB/AM | Incubating for 7d without changing the medium to deplete the serum | (Xiang et al., 2022) |
|  |  |  | DMEM+HEPES+ F-10 Nut Mix containing 10 % FBS, 1% MEM-non essential amino acids, 1% GlutaMAX, 1% AB/AM, 50 μM ZnCl_2_, 100 ng/ml IGF-1, 62 ng/ml HB-EGF | DMEM supplemented with 2% HS, 1% AB/AM, 100 ng/ml IGF-1 and 62 ng/ml HB-EGF | (Zagury et al., 2022) |
|  |  | MSCs | LG-DMEM supplemented with 10% FBS and 1% AB/AM | LG-DMEM supplemented with 5% FBS, 10 μM insulin, 1 μM DEX, 10 μM ciglitizone, and 100 μM oleic acid. | (Lee et al., 2024b) |
|  |  |  | α-Minimal Essential Medium(α-MEM) containing 10 % FBS, 1% P/S, 0.8% AmB, and 0.25 ng/ml bFGF |  | (Yen et al., 2023) |
|  |  |  | α-MEM containing 10 % FBS and 1% AB/AM |  | (Zernov et al., 2022) |
|  |  |  | IMEM containing 10 % FBS | IMEM containing 10 % FBS, pristanic acid, phytanic acid, erucic acid, elaidic acid, oleic acid, palmitoleic acid and myristoleic acid | (Louis et al., 2023) |
|  |  |  | HG-DMEM containing 10 % FBS | HG-DMEM containing 10 % FBS, Rock-inhibitor, WNT inhibitor and bFGF | (Zagury et al., 2022) |
|  |  |  |  | DMEM/F12 supplemented with 135nM transferrin, 1.8μM insulin and 10 µM lysophosphatidic acid | (Messmer et al., 2022) |
| **Refer to SUPPLEMENTAY 3 (continued)** | | | | | |
| **Class** | **Genus** | **Seed cells** | **Growth Medium** | **Differentiation Medium** | **Reference** |
| Mammalia | Porcine | Myoblasts | F10 containing 10% FBS, 10ng/ml bFGF and 1% P/S | HG-DMEM supplemented with 2% HS and 1% P/S | (Kong et al., 2023b) |
|  |  | MuSCs | DMEM/F-12 containing 15 % FBS, 15ng/ml bFGF and 1% P/S | DMEM supplemented with 2% HS and 1% P/S | (Ding et al., 2023) |
|  |  |  | DMEM containing 10 % FBS and 1 % P/S | DMEM supplemented with 2% HS, BSA, AAS and ITS supplement and naringenin. | (Guan et al., 2023) |
|  |  |  | Growth Medium-2 BulletKit spplemented with 1 × Glutamax, 1 × AB/AM, 0.1 mM β-mercaptoethanol and 20 μM SB203580 | Welgene medium containing 1 × Glutamax, 1 × Antibiotic-antimycotic, 0.1 mM β-mercaptoethanol, and 1% KnockOut™ Serum Replacement | (Kim et al., 2023) |
|  |  | SMCs | DMEM/F12 containing 10 % FBS and 1 % P/S | DMEM/F12 containing DEX, IBMX, rosiglitazone and indomethacin. | (Zheng et al., 2022a) |
|  |  |  | DMEM/F12 containing 15 % FBS, 5 ng/ml bFGF and 1% P/S |  | (Zheng et al., 2022b) |
|  |  |  | DMEM/F12 containing 20 % FBS and 1 % P/S |  | (Zheng et al., 2021) |
|  |  | pre-adipocytes | DMEM supplemented with 15% FBS | DMEM supplemented with 0.1 mM IBMX, 1 μM DEX, 10 μg/mL insulin, and 10 μM rosiglitazone. | (Gu et al., 2024) |
|  |  | MSCs | DMEM/F12 containing 10 % FBS and 5ng/ml bFGF and 1% P/S | (D0-D5) DMEM/F12 containing 10 % FBS, 1μM DEX,10μg/ml insulin,0.1mM IBMX, 1μM rosiglitazone,0.1 mM indomethacin and 1 % P/S (D5-D7) DMEM/F12 containing 10 % FBS, 5ng/ml bFGF and 1 % P/S (D7-D12) DMEM/F12 containing 10 % FBS and 1 % P/S | (Song et al., 2022a) |
|  |  |  | DMEM/F12 containing 10 % FBS and 1% P/S | DMEM/F12 containing 10 % FBS, 1% P/S, 10 ng/mL insulin, 1 mM DEX, 0.1 mM IBMX and 1 nM rosiglitazone | (Chen et al., 2023a) |
| **Refer to SUPPLEMENTAY 3 (continued)** | | | | | |
| **Class** | **Genus** | **Seed cells** | **Growth Medium** | **Differentiation Medium** | **Reference** |
| Mammalia | Rabbit | Myoblasts | DMEM/F12 containing 20 % FBS, and 1% P/S | DMEM/F12 supplemented with 2% HS and 1% P/S | (Zernov et al., 2022) |
|  |  | SMCs | HG-DMEM containing 10 % FBS and 1% AB/AM | - | (Zernov et al., 2022) |
|  |  | pre-adipocytes | DMEM containing 20 % FBS | DMEM supplemented with 10 % FBS, 0.25 mM DEX, 10 μg/ml insulin, 0.5mM IBMX and 0.2 mM ascorbic acid 2-phosphate | (Kawecki et al., 2023) |
|  | Mouse | C2C12 | DMEM containing 10 % FBS and 1 % PS | DMEM supplemented with 2 % HS and 1 % P/S | (Chen et al., 2024b; Kong et al., 2023b; Norris et al., 2022; Yamanaka et al., 2023) |
|  |  |  | DMEM containing 20 % FBS and 1% P/S | DMEM supplemented with 2 % HS and 1 % P/S | (Chen et al., 2023b; Park et al., 2023) |
|  |  |  | DMEM containing 10 % FBS and 1% AB/AM | Incubating for 7 days without changing the medium to deplete the serum | (Xiang et al., 2022) |
|  |  |  | DMEM containing 10 % FBS, 14.3/25/100 μg/ml C-phycocyanin and 1 % P/S | - | (Park et al., 2021) |
|  |  | 3T3-L1 | HG-DMEM containing 10% FBS, and 1% P/S | HG-DMEM supplemented with 0.5 mM IBMX, 0.25 µM DEX, 1 µg/mL insulin, and 2 µm rosiglitazone | (Kong et al., 2023b) |
|  |  |  | DMEM containing 10% super calf serum | DMEM supplemented with 10 % FBS, 0.25 mM DEX, 10 μg/ml insulin, 3-IBX and 0.2 mM ascorbic acid 2-phosphate | (Kawecki et al., 2023) |
|  | Ovine | Fibroblasts | HG-DMEM containing 10% FBS and 1% AB/AM | - | (Zernov et al., 2022) |

| **Refer to SUPPLEMENTAY 3 (continued)** | | | | | |
| --- | --- | --- | --- | --- | --- |
| **Class** | **Genus** | **Seed cells** | **Growth Medium** | **Differentiation Medium** | **Reference** |
| Aves | Chicken | Myoblasts | DMEM/F12 containing 15% FBS and 100ng/ml recombinant human bFGF | HG-DMEM supplemented with 10% HS, 5% chick embryo extract with 10% Matrigel | (Hong & Do, 2024) |
|  |  | MuSCs | DMEM/F12 containing 20% FBS and 1% P/S | DMEM/F12 supplemented with 2% HS and 1% P/S | (Chen et al., 2023b) |
|  |  | Spontaneously immortalized embryonic fibroblasts | DMEM/F12 containing 3 µg/ml insulin, 10 ng/ml bFGF, 2 µg /ml hydrocortisone, 7 ng/ml sodium selenite, 2 mM L-alanine-L-Glutamine and 10 µg/ml canola lipid mixture | DMEM supplemented with 10% FBS, 2 mM L-alanine-L-Glutamine, 200 µM Oleic acid and 10μM rosiglitazone, 50 µM pristanic acid or 12 µg/ml L-α-Phosphatidylcholine) | (Pasitka et al., 2023) |
| Pisce | Fish | MuSCs | HG-DMEM containing 15% FBS, 10 ng/ml bFGF, 200 μM Pifithrin-α hydrobromide and 1X P/S | F12 medium containing 8% HS, 10 ng/ml IGF-1, 50 nM necro sulfonamide, 200 μM ascorbic acid and 1X P/S | (Xu et al., 2023) |
|  |  | MSCs | HG-DMEM containing 8% FBS, 10 ng/ml bFGF, 200 μM Pifithrin-α hydrobromide and 1X P/S | DMEM/ F12 containing 10% HS, 10 μg/mL insulin, 0.5 μM IBMX, 0.25 μM DEX, 1% Lipid Mixture and 1 × P/S | (Xu et al., 2023) |

**Abbreviations**: MuSCs, Muscle Stem Cells; MSCs, Mesenchymal Stem Cells; SMCs, Smooth Muscle Cells; DMEM, Dulbecco's Modified Eagle Medium; LG-DMEM, Low-Glucose Dulbecco’s Modified Eagle’s Medium; HG-DMEM, High-Glucose Dulbecco’s Modified Eagle’s Medium; AB/AM, Antibiotic–Antimycotic; FBS, Fetal Bovine Serum; HS, Horse Serum; P/S/G, Penicillin-Streptomycin-Glutamine; HEPES, 4-(2-Hydroxyethyl)-1-Piperazineethanesulfonic Acid; IGF-1, Insulin-Like Growth Factor; HB-EGF, Heparin-Binding Epidermal Growth Factor-Like Growth; DEX, Dexamethasone; Α-MEM, Α-Minimal Essential Medium; AmB, Amphotericin B; bFGF, Basic Fibroblast Growth Factor; IMEM, Iscove's Modified Dulbecco's Medium; BSA, Bovine Serum Albumin; AAS, MEM Amino Acid Solution; ITS, Insulin-Transferrin-Selenium; IBMX, 3-Isobutyl- 1-Methylxanthin
